# Topical and Scaffold-Mediated Delivery of VCE-005.1 Enhances Diabetic Wound Healing Through Endothelial Protection and Vascular Regeneration

**DOI:** 10.3390/pharmaceutics18101200

**Published:** 2026-09-22

**Authors:** Isabel Lastres-Cubillo, María E. Prados, Esther Rincón, Eduardo Espinosa, Juan Domínguez-Robles, Ilyas Essadki-Aittaji, Adela García-Martín, Eduardo Muñoz

**Affiliations:** 1 Maimonides Biomedical Research Institute of Córdoba (IMIBIC), 14004 Córdoba, Spain; isabel.lastres@imibic.org (I.L.-C.); mariaeugenia.prados@gmail.com (M.E.P.); 2 Hospital Universitario Reina Sofía, 14004 Córdoba, Spain; 3 Cellular Biology, Physiology, and Immunology Department, University of Córdoba, 14014 Córdoba, Spain; 4 BioPrEn Group (RNM940), Chemical Engineering Department, Instituto Químico para Energía y el Medioambiente (IQUEMA), Faculty of Science, University of Córdoba, 14014 Córdoba, Spain; b32rirue@uco.es (E.R.); a02esvie@uco.es (E.E.); 5 Department of Pharmacy and Pharmaceutical Technology, Faculty of Pharmacy, Universidad de Sevilla, 41012 Seville, Spain; jdominguez6@us.es (J.D.-R.); iessadki@us.es (I.E.-A.)

**Keywords:** B55α activators, HIF-1α, AMPK, SIRT1, angiogenesis, diabetic wound healing

## Abstract

**Background/Objectives**: Chronic diabetic wounds remain a major clinical challenge due to persistent inflammation, endothelial dysfunction, impaired angiogenesis, and defective tissue regeneration. Local therapies that restore vascular repair while providing sustained drug delivery are urgently needed. This study investigated the therapeutic efficacy of VCE-005.1, a hypoxia-mimetic betulinic acid derivative, administered topically or incorporated into a biocompatible and biodegradable polycaprolactone (PCL) scaffold, and examined the molecular mechanisms underlying its pro-regenerative activity. **Methods**: Endothelial protective activity was evaluated in endothelial cells exposed to hyperglycemic and oxidative stress by assessing cell viability, wound healing, senescence, and engagement of a B55α-associated signaling network involving AMPK, SIRT1, and HIF-1α. A VCE-005. 1-loaded PCL scaffold was fabricated and characterized for physicochemical properties, biological activity, and fibroblast adhesion. Therapeutic efficacy was then evaluated in excisional wound-healing models using diabetic (db/db) and non-diabetic (db/+) mice following topical or scaffold-mediated treatment. **Results**: VCE-005.1 protected endothelial cells against hyperglycemic and oxidative stress by enhancing AMPK phosphorylation, increasing SIRT1 expression and activity, stabilizing HIF-1α, and reducing endothelial senescence. The PCL scaffold preserved the biological activity of VCE-005.1 and enabled its local delivery. Both topical and scaffold-mediated administration significantly accelerated wound closure, improved histological wound architecture, enhanced angiogenesis and vascular maturation, restored endothelial SIRT1 expression in vivo, and reduced macrophage (F4/80^+^) and neutrophil (MPO^+^) infiltration. **Conclusions**: Topical and scaffold-mediated delivery of VCE-005.1 markedly improves wound repair through endothelial protection, vascular regeneration, and attenuation of local inflammation. These findings support VCE-005.1 as a promising local therapy for diabetic foot ulcers and support biodegradable scaffold-based delivery to enhance its therapeutic potential.

## 1. Introduction

Diabetic foot ulcers (DFU) remain a major clinical challenge due to their chronicity, high recurrence rates, and the risk of lower-limb amputation. These ulcers develop in approximately 15–25% of diabetic patients over their lifetime and are frequently associated with vascular insufficiency and neuropathy. Impaired blood flow, endothelial dysfunction, and defective tissue repair lead to chronic peripheral tissue hypoxia, creating a hostile wound environment that delays healing, promotes infection, and increases morbidity and healthcare costs [1,2,3]. These challenges highlight the urgent need for new therapeutic strategies capable of restoring endothelial function and supporting functional vascular regeneration, ultimately promoting effective wound healing in DFU.

In DFU, ischemia and hypoxia activate compensatory pro-angiogenic pathways, primarily through HIF-1α and growth factors like vascular endothelial growth factor (VEGF). However, persistent endothelial dysfunction, driven by oxidative stress and chronic inflammation, disrupts these repair signals. As a result, even with increased VEGF levels, the damaged vascular environment fails to support proper blood vessel formation, leading to inadequate perfusion and poor tissue regeneration. This dysfunctional endothelium remains a key obstacle to achieving effective therapeutic angiogenesis in DFUs [4,5].

HIF-1α is a key transcriptional regulator of the cellular response to hypoxia, orchestrating the expression of pro-angiogenic factors such as VEGF, hepatocyte growth factor (HGF), and erythropoietin (EPO). Under hypoxic conditions, inhibition of prolyl hydroxylases (PHDs) promotes HIF-1α stabilization and activation of genes essential for angiogenesis and vascular remodeling [6]. Accordingly, hypoxia-mimetic strategies, including controlled exposure to low oxygen or pharmacological PHD inhibition, have emerged as promising approaches to enhance HIF-1α signaling, vascular repair, and tissue regeneration, although their application in DFU remains underexplored [7,8].

SIRT1, a NAD^+^-dependent deacetylase, has emerged as a critical regulator of endothelial function and vascular homeostasis under metabolic stress conditions. In diabetic conditions, reduced SIRT1 expression has been associated with endothelial senescence, impaired nitric oxide bioavailability, increased oxidative stress, and microvascular rarefaction [9,10]. Restoration of SIRT1 signaling improves angiogenic responses, attenuates inflammation, and enhances tissue repair [11]. In addition, SIRT1 regulates mitochondrial function and redox homeostasis, processes that are compromised in diabetic endothelium and are essential for cellular adaptation to hypoxic stress [12].

Importantly, SIRT1 function is closely linked to AMP-activated protein kinase (AMPK), a central metabolic sensor that coordinates cellular energy balance with endothelial survival and angiogenesis. AMPK activation promotes endothelial survival, nitric oxide production, and angiogenesis, while also enhancing SIRT1 activity through modulation of the NAD^+^ pool [13]. Dysregulation of the AMPK/SIRT1 axis has been implicated in defective vascular repair and chronic inflammation in diabetic wounds, supporting the therapeutic rationale for targeting this pathway in DFU [12,14,15].

Recent research suggests that B55α (PPP2R2A), a regulatory subunit of protein phosphatase 2A (PP2A), may represent a novel pharmacological target in vascular diseases. The B55α subunit dephosphorylates prolyl hydroxylase-2 (PHD2) at Ser125, reducing its activity and thereby promoting HIF-1α stabilization [16]. Additionally, B55α promotes endothelial cell (EC) stability, protecting them against oxidative stress and apoptosis during vascular remodeling [17]. This mechanism not only preserves endothelial integrity but also fosters angiogenesis, supporting new blood vessel formation. On the other hand, SIRT1 and AMPK are complementary regulators of endothelial metabolism and vascular repair, whose dysregulation contributes to impaired angiogenesis in DFU [12,14,15], further supporting the therapeutic potential of compounds targeting the AMPK/SIRT1 axis.

Given the role of PP2A/B55α-associated signaling in vascular homeostasis, compounds targeting this pathway, such as VCE-005.1, emerge as promising candidates for therapeutic intervention in DFU. VCE-005.1, a betulinic acid hydroxamate currently in preclinical development, is a post-translational PHD2 inhibitor that modulates PP2A/B55α-associated signaling [18]. It has demonstrated efficacy in various preclinical models, including neonatal intraventricular hemorrhage [19], hypoxic–ischemic brain injury [20], inflammatory bowel disease [21], and traumatic brain injury (TBI) [22].

To improve the local delivery of pharmacological agents targeting vascular repair pathways, recent advances in tissue engineering have highlighted the potential of 3D-scaffolds for the treatment of DFU. These biomaterials, fabricated from natural or synthetic polymers, provide structural support while enabling localized and sustained drug delivery, thereby improving angiogenesis and tissue regeneration compared to conventional dressings [23,24,25,26]. Among synthetic polymers, polycaprolactone (PCL) has gained particular attention due to its biocompatibility, mechanical stability, and controlled degradation profile, making it suitable for drug-loaded scaffolds designed to enhance vascularization and wound repair in preclinical models [23,26,27].

While B55α has been linked to hypoxic signaling through the PHD2/HIF-1α axis, whether this pathway integrates metabolic stress sensors such as AMPK and SIRT1 during endothelial repair remains unclear. In this study, we investigated the effects of VCE-005.1 on the B55α-associated AMPK/SIRT1/HIF-1α signaling network and evaluated its therapeutic efficacy in preclinical models of wound healing using both topical administration and PCL-based scaffolds for local drug delivery. We further assessed its effects on endothelial protection, vascular regeneration, tissue repair, and local inflammation. These findings provide a preclinical rationale for the development of VCE-005.1 as a local therapeutic strategy for DFU.

## 2. Materials and Methods

### 2.1. Cell Cultures and Reagents

Human neonatal dermal fibroblasts (Innoprot, Derio, Spain), which play a key role in extracellular matrix production and wound remodeling, were similarly immortalized by SV40 overexpression to establish the FIBSV40 line, which was subsequently modified by lentiviral transduction with a construct encoding a nuclear-localized green fluorescent protein (GFP) to generate FIBSV40-GFP. Lentiviral transduction was performed using the Incucyte^®^ Nuclight Green Lentivirus 2.0 (bleo) kit (BA-04890, Sartorius, Germany), following the manufacturer’s instructions.

EA.hy926 cells (ATCC^®^ CRL-2922™), a human endothelial cell line derived from the fusion of HUVECs with the A549 cell line, were used as a model of vascular endothelium to evaluate endothelial viability and wound healing responses. EA.hy926 cells were used at passages below 10 for all experiments. NIH-3T3-EPO-Luc cells, a murine fibroblast reporter cell line stably transfected with an EPO promoter-driven luciferase construct [28] (Minassi et al., 2018), were used to evaluate HIF-1α transcriptional activity. Human microvascular endothelial cells (HMEC-1) (ATCC^®^ CRL-3243™), a human microvascular endothelial cell line, were used to study endothelial responses under stress conditions.

All cells except HMEC-1 were maintained in DMEM (Pan-Biotech, Aidenbach, Germany) supplemented with 10% FBS and 1% (*v*/*v*) penicillin/streptomycin, whereas HMEC-1 cells were cultured in MCDB131 medium (Pan-Biotech) supplemented as previously described in [29].

Cell lines were incubated at 37 °C in a humidified atmosphere containing 5% CO_2_ and were routinely tested and confirmed to be mycoplasma-free throughout the course of the experiments. Cell line authentication was performed by the original suppliers or ATCC, and none of the cell lines used in this study have been reported as misidentified or contaminated.

VCE-005.1 (99.1% purity) was provided by VivaCell Biotechnology España S.L. (Córdoba, Spain) and dissolved as reported by [7,21]. The chemical structure of VCE-005.1 has been reported previously by [21].

### 2.2. siRNA Transfections and Luciferase Assays

The RNAi assays were conducted using ON-TARGETplus SMARTpools targeting SIRT1 (#L-003540) and B55α (#L-004824), along with a non-specific siRNA control pool (#D-001810) (Dharmacon, Lafayette, CO, USA). A concentration of 10 nM was used in all cases. For luciferase assays, the NIH-3T3-EPO-luc cells (10^4^ cells/well) were seeded in 96-well plates. After 24 h, the cells were pre-treated with the inhibitors for 30 min and then exposed to the compound VCE-005.1 for 6 h. The inhibitors used included Dorsomorphin (DS) (AMPK inhibitor #S7306, Selleckchem, Houston, TX, USA), EX527 (SIRT1 inhibitor #E7034, Sigma-Aldrich, St. Louis, MO, USA), and CCT018159 (HSP90 inhibitor #7687880, Sigma-Aldrich). After treatment, cells were lysed in 50 µL lysis buffer containing 25 mM Trisphosphate (pH 7.8), 8 mM MgCl_2_, 1 mM DTT, 1% Triton X-100, and 7% glycerol. Using a TriStar2 Berthold/LB942 multimode reader (Berthold Technologies, Bad Wildbad, Germany), luciferase activity was measured according to the instructions provided by the luciferase assay kit (Promega, Madison, WI, USA).

### 2.3. Western Blots

After stimulation and washing with phosphate-buffered saline (PBS), proteins were harvested using 50 μL of RIPA buffer containing phosphate and protease inhibitors (50 mM Tris-HCl, pH 7.5, 150 mM NaCl, 2 mM EDTA, 1% NP40, 0.5% sodium deoxycholate, 0.5 mM Na_3_VO_4_, 50 mM NaF, and 40 μg/μL Protease Inhibitor Cocktail) or with lysis buffer previously described by [18]. Proteins (40 μg) were heated at 95 °C in Laemmli buffer and separated by electrophoresis on 8–12% SDS-PAGE gels. Subsequently, proteins were transferred onto nitrocellulose membranes for 30 min at 24 V. The membranes were then blocked with tris-buffered saline (TBS) containing 0.1% Tween 20 and 5% non-fat dry milk for 1 h RT. Immunodetection of specific proteins was performed by incubating the membranes with the primary antibody overnight at 4 °C (Table S1). After washing, the membranes were incubated with a secondary antibody (Table S1) for 1 h and detected using the ChemiDoc MP Imaging System (Bio-Rad, Hercules, CA, USA). Protein expression levels were normalized to β-actin, except for pAMPK and peNOS, which were normalized to their corresponding total protein levels (AMPK and eNOS, respectively). All experiments were performed in triplicate.

### 2.4. SIRT1 Activity Assay

The SIRT1 activity assay was conducted using the fluorometric SIRT1 Activity Assay Kit (#ab156065, Abcam, Cambridge, UK), according to the manufacturer’s instructions. Fluorescence was measured over time, and SIRT1 activity was calculated as the slope of the linear portion of the fluorescence curve (RFU/min) after subtraction of background signal obtained from the no-enzyme control.

### 2.5. NAD^+^/NADH Assay

The NAD^+^/NADH assay was performed using the NAD^+^/NADH assay kit (#ab65348, Abcam), following the manufacturer’s instructions. EA.hy926 cells (2 × 10^6^ cells/well) were seeded and stimulated for 4 h before measuring NAD^+^/NADH levels.

### 2.6. Senescence Assay

HMEC-1 cells were incubated with different concentrations of VCE-005.1 for one hour and subsequently treated with H_2_O_2_ for 4 h. This treatment was repeated on days 2 and 5. At day 7, and following the manufacturer’s instructions, Senescence-Associated β-Galactosidase (SA-β-gal) staining (#9860, Cell Signaling Technology, Danvers, MA, USA) was performed.

### 2.7. Cell Viability Under High Glucose Conditions

EA.hy926 cells (1 × 10^4^ cells/well) were seeded in 96-well plates and cultured for 24 h in complete medium. Cells were then exposed to high-glucose conditions (30 mM or 80 mM D-glucose; Sigma-Aldrich) for 4 days, with medium renewal during this period.

These glucose concentrations were selected as experimental conditions commonly used to induce different degrees of high-glucose-associated cellular stress and cytotoxicity, allowing the protective effects of VCE-005.1 to be evaluated under moderate and more stringent conditions. After glucose preconditioning, cells were treated with increasing concentrations of VCE-005.1 for 24 h. MTT (5 mg/mL; 3-(4,5-dimethylthiazol-2-yl)-2,5-diphenyltetrazolium bromide; CT01-5, Sigma-Aldrich) was then added and incubated for 4 h. The medium was removed, and the formazan crystals were dissolved in DMSO. Absorbance was measured at 550 nm using a microplate reader (Tecan, Männedorf, Switzerland). Cell viability was expressed as a percentage relative to the control group.

### 2.8. In Vitro Wound Healing Assay

EA.hy926 cells were seeded in 96-well ImageLock plates (Essen BioScience, Ann Arbor, MI, USA) at a density of 2 × 10^4^ cells/well and maintained at 37 °C in a humidified CO_2_ incubator. After 24 h, cells were exposed to high-glucose conditions (30 or 80 mM D-glucose) for an additional 24 h to mimic hyperglycemic stress. Wounds were generated using a 96-pin WoundMaker (Essen BioScience, Ann Arbor, MI, USA), and cells were washed to remove debris before incubation in incomplete medium in the presence or absence of VCE-005.1. Wound images were acquired at 0 and 24 h, and wound closure was quantified using Fiji software (ImageJ version 1.54f, National Institutes of Health, Bethesda, MD, USA).

### 2.9. Fabrication of PCL 3D-Scaffolds

For the fabrication of the PCL-based scaffolds, two different forms of PCL, CAPA™ 6506 (*M*_W_ = 50,000 Da, i.e., high molecular weight), henceforth referred to as H-PCL, and CAPA™ 2054 (*M*_W_ = 550 Da, i.e., low molecular weight), henceforth referred to as L-PCL, were both provided by Ingevity (Warrington, UK).

Prior to 3D printing, H-PCL was heated at 70 °C for 2 h until a homogeneous and transparent melt was achieved. The molten H-PCL was then transferred into a cylindrical syringe and allowed to cool and solidify. Once cooled, the resulting cylindrical preform was removed from the syringe using compressed air, thereby obtaining a cylindrical PCL preform suitable for subsequent loading into the stainless-steel cartridge of the thermoplastic printhead. For unloaded scaffolds, the preforms contained 5 g of PCL.

For PCL-based scaffolds containing the bioactive compound VCE-005.1, a nominal loading of 1% *w*/*w* VCE-005.1 relative to the total preform mass was used. For a 5 g batch, 0.05 g of VCE-005.1 was first weighed and mixed with 0.10 g of L-PCL at 70 °C under continuous stirring. Separately, 4.85 g of H-PCL was heated at 70 °C for 2 h until a homogeneous and transparent melt was obtained. The molten H-PCL was then transferred to the vessel containing the VCE-005.1/L-PCL mixture. The resulting composite was maintained at 70 °C for 1 h, with intermittent manual mixing to promote homogeneous dispersion of VCE-005.1 throughout the PCL matrix. The final composite was subsequently transferred into a cylindrical syringe, and the preforms were then produced as described above. The final printed scaffolds weighed approximately 30 mg, corresponding to approximately 300 µg of VCE-005.1 per scaffold based on the nominal 1% (*w*/*w*) loading.

Before printing, the cylindrical PCL preforms were loaded into the stainless-steel cartridge of a thermoplastic printhead (Cellink, Gothenburg, Sweden) and preheated at 120 °C for 15 min. Cylindrical scaffolds (5 mm height, single layer) were designed using computer-aided design (CAD) software and printed with a 3D bioprinter (BioX6, Cellink). Printing parameters were set as follows: nozzle size, 25 G (0.25 mm inner diameter) with 32 mm length; printing temperature, 120 °C; build plate temperature, 60 °C; flow rate, 4 mm/s; infill density, 30% with grid pattern; patch diameter, 10 mm; pressure, 200 kPa.

### 2.10. Physicochemical and Mechanical Characterization of PCL-Based 3D-Scaffolds

The incorporation of the bioactive compound VCE-005.1 in PCL matrices was evaluated by Fourier-transform infrared (FTIR) spectroscopy using a Spectrum Two FT-IR Spectrometer (Perkin Elmer, Waltham, MA, USA) equipped with the attenuated total reflectance (ATR) accessory. The spectrum was analyzed with a resolution of 4 cm^−1^ in the range of 4000–450 cm^−1^. A total of 20 scans were collected for each sample. The spectra were baseline corrected and subsequently normalized prior to graphical representation to facilitate comparison between the samples.

The wettability of the scaffolds was quantified by measuring the water contact angle (WCA) on a contact angle goniometer (Ossila, Sheffield, UK) using the sessile drop method. A 10 µL drop of distilled water was applied to the surface of the scaffolds, and the angle was measured over a period of 0 to 10 s. The measurements were repeated five times, and all results were expressed as the mean value with its standard deviation.

Mechanical properties of the 3D scaffolds were evaluated by compression tests using a Universal Testing Machine model LF Plus (Lloyd Instrument AMETEK Measurement & Calibration Technologies Divion, Largo, FL, USA) with a 1kN load cell. Tests were carried out at 2 mm/min at room temperature. As a result, stress–strain curves were obtained from strain values up to the 50% strain limit. The slope of the linearized stress–strain data was used in the final estimates of the moduli. The analyses were performed in triplicate, and the results are reported as the mean value and its standard deviation.

### 2.11. Bioactivity Characterization of 3D-Scaffolds

To evaluate the biological activity of VCE-005.1 contained in the scaffolds, the constructs were incubated in DMEM in 24-well plates, and conditioned media were collected at the indicated time points. NIH-3T3-EPO-luc cells (7 × 10^4^ cells/well) previously seeded in 24-well plates were then exposed to the collected conditioned media for 24 h. Luciferase activity was subsequently determined as described above and expressed as fold induction relative to control 3D-scaffolds, which were set to 1.

For cell adhesion studies, FIBSV40-GFP fibroblasts (2 × 10^4^ cells/well) were seeded onto control or VCE-005.1-loaded 3D-scaffolds placed in 24-well plates. Fluorescence images were acquired at the indicated time points using a Thunder Imager 3D fluorescence microscope (Leica Microsystems, Madrid, Spain) at ×5 magnification. Three independent patches per condition were analyzed, with 3–4 images acquired from each scaffold and averaged to obtain a single value per scaffold. Cell adhesion was quantified by determining the density of adherent cells relative to the scaffold area at each time point.

### 2.12. Animals

Mice used in the study were sourced from Charles River Laboratories (Barcelona, Spain) and Janvier Labs (Le Genest-Saint-Isle, France). Animals were housed with unrestricted access to standard food and water in a controlled environment (temperature 20 °C (±2 °C), 40–50% relative humidity, and a 12 h light/dark cycle).

All procedures were approved by the institutional ethics committee for the care and use of animals. The experiments were conducted following the guidelines of animal care set by the European Directive 2010/63 and the corresponding Spanish regulations (RD 53/2013 and Order ECC/566/2015). The Ethics Committee on Animal Experimentation at the University of Córdoba (Spain) and the Andalusian Regional Committee for Animal Experimentation approved all procedures described in this study (Project Numbers 01/06/2020/072 and 23/05/2024/068). Animal numbers were determined in accordance with the principles of the 3Rs (Replacement, Reduction, and Refinement), balancing ethical considerations with the need to obtain statistically meaningful results. Two complementary in vivo models (topical formulation and scaffold-based delivery) were employed, and control groups were minimized and shared whenever possible to reduce animal use. All efforts were made to minimize animal suffering.

### 2.13. In Vivo Wound Healing Experiments

Male 8-week-old diabetic db/db mice and their normoglycemic heterozygous db/+ controls were used for in vivo wound healing studies. Two complementary experimental models were performed to evaluate the effect of VCE-005.1 treatment, delivered either in a topical cream or incorporated into 3D-scaffolds.

Animals were randomly assigned to the experimental groups. Investigators performing wound measurements, histological scoring, and image quantification were blinded to treatment allocation. No predefined exclusion criteria were applied.

For the topical treatment model, mice were divided into four experimental groups: db/+ + vehicle (n = 4), db/+ + VCE-005.1 (n = 5), db/db + vehicle (n = 4), and db/db + VCE-005.1 (n = 5). On the day of injury, the mice were anesthetized with isoflurane, and their dorsal hair was removed using an electric razor and depilatory cream. A silicone rubber splint (internal and external diameters of 8 and 15 mm, respectively) was placed around each wound to prevent wound contraction. The splint was secured using a small amount of 3M Vetbond Tissue Adhesive (n-butyl cyanoacrylate; catalog no. 1469SB; 3M, St. Paul, MN, USA), applied to the surface of the splint that was placed in contact with the intact periwound skin, and fixed with 4–6 interrupted sutures. Care was taken to avoid any contact between the adhesive and the wound bed or exposed tissue. 50 µL of VCE-005.1 treatment (cream containing 1% of compound, 5% liquid petrolatum, and 94% solid petrolatum) or vehicle consisting of the same formulation without active compound (cream with 5% liquid petrolatum and 95% of solid petrolatum) was applied topically to the wound site every day using a syringe, and a transparent bandage (Tegadem 3 M) was used to cover the wound. Digital photographs were taken every two to three days over a period of 10 days.

For the scaffold-based model, mice were also divided into four experimental groups: db/+ with control scaffold (n = 5), db/+ with VCE-005.1 3D-scaffold (n = 6), db/db with control 3D-scaffold (n = 5), and db/db with VCE-005.1 3D-scaffold (n = 6). Wounds were created as described above. In this model, silicone splints were not used to allow blood flow measurements. The corresponding 3D-scaffolds were placed directly into the wound site and left in place until day 14. Digital photographs and non-invasive blood flow assessment were acquired simultaneously every 2–3 days using the moorFLPI speckle contrast imaging system (Moor Instruments, Madrid, Spain).

Two wounds were created in each animal, and both received the same treatment. For wound-closure and perfusion analyses, both wounds were evaluated, and their values were averaged at each time point to obtain a single value per animal. At the study endpoint, one wound was used for histological analyses and the other for molecular analyses. Thus, the animal was considered the experimental unit.

Wound areas were measured in pixels using different approaches for each model. For the topical cream model, measurements were performed using a ruler and the silicone splint, and the analysis was performed using ImageJ 1.32 software (National Institutes of Health, Bethesda, MD, USA). In the 3D-scaffold model, wound areas were determined using MoorFLPI-2 Software Review 6.0 (Moor Instruments, Axminster, UK) as a reference. Results were expressed as the percentage of wound closure at each evaluation time point. The primary outcome measure in both wound-healing studies was the percentage of wound closure over time. Secondary outcome measures included tissue perfusion, histological wound healing score, vascular regeneration, inflammatory cell infiltration, and molecular analyses of wound tissue.

### 2.14. Blood Perfusion Analysis

Perfusion images obtained immediately after wound creation (day 0) and on days 3, 7, 10, and 14 were analyzed using moorFLPI-2 Review Software V6.0. For each wound, a circular ROI with a fixed diameter of 8 mm was placed over the wound site. The wound bed was delineated at each time point and excluded from this ROI, allowing the mean flux in the surrounding periwound tissue to be determined. The 8 mm diameter was chosen to include the initial 6 mm wound together with a margin of surrounding tissue. The same procedure was followed for all animals, experimental groups, and time points.

A second circular ROI, also 8 mm in diameter, was placed on an area of healthy, unwounded skin from the same animal, using the same selection criteria across all images. Mean flux values for the periwound area and healthy skin were recorded in perfusion units (PU). For each wound, the periwound value was normalized to the healthy-skin value obtained from the same animal and time point and expressed as relative blood flow versus healthy skin. This ratio was dimensionless. As two wounds were created in each mouse, the normalized values from both wounds were averaged at each time point to obtain a single value per animal. The animal was therefore considered the experimental unit.

### 2.15. Histology, Immunohistochemistry and Immunofluorescence

Paraffin sections (5 μm) were processed and examined using H&E staining or incubated with Harris Hematoxylin, Acid Fuchsine, Phosphomolybdic acid, and 1% Acetic acid for Masson’s Trichrome staining. Histological evaluation was performed using a semi-quantitative scoring method, as previously described [30]. Histological scoring was performed using all available animals.

For immunohistochemical (IHC) analysis, tissue sections (5 µm thick) were deparaffinised and subjected to boiling treatment in 1× citrate buffer for 10 min, followed by incubation in a solution of 3.3% H_2_O_2_ in methanol for 10 min. Subsequently, the samples were washed three times with PBS containing 0.1% Tween, treated with a blocking solution (#20773, Merck, Darmstadt, Germany) and left to incubate at 4 °C overnight with the corresponding primary antibody (Table S1). After that the slides were incubated at 1 h RT with the appropriate secondary antibody (Table S1). Following this, the samples were treated with avidin-biotin-peroxidase reagent (PK-6100, Vector Laboratories, Newark, CA, USA) and DAB (3,3′-diaminobenzidine) chromogen (K346811-2, Dako, Santa Clara, CA, USA). The preparations were then counterstained with hematoxylin followed by dehydration and mounted with Eukitt mounting medium.

For immunofluorescence analysis, tissue sections were processed as described above and incubated overnight at 4 °C with the corresponding primary antibodies (Table S1), followed by incubation with the appropriate fluorophore-conjugated secondary antibodies for 1 h at room temperature in the dark. Sections were mounted with DAPI-containing medium and visualized using an LSM710 spectral confocal laser-scanning microscope (Carl Zeiss, Jena, Germany). Images used for quantitative comparisons were acquired using identical confocal settings within each staining experiment. Background fluorescence was corrected in ImageJ using the Subtract Background function with the same parameters for all images within each analysis.

For immunohistochemistry and immunofluorescence analyses, four animals per group were randomly selected, independently of the experimental results, to maintain the same sample size across groups. For each animal, 4–5 fields from different areas of the wound tissue were analyzed and averaged to obtain a single value per animal. Thus, each animal contributed one value to the statistical analysis. Depending on the marker, immunofluorescence was quantified as background-corrected integrated density, the number of marker-positive or double-positive cells or vessels normalized to the analyzed tissue area, vessel lumen area relative to the total tissue area analyzed, or vessel lumen perimeter. Endoglin^+^ and CD31^+^/α-SMA^+^ vessel densities were calculated as the number of positive vessels divided by the corresponding tissue area and expressed as vessels/µm^2^. Nuclear HIF-1α localization was determined as the percentage of HIF-1α^+^/DAPI^+^ nuclei relative to the total number of DAPI^+^ nuclei.

### 2.16. RNA Extraction and qRT-PCR

For qPCR analyses, four animals per group were randomly selected, independently of the experimental results, to maintain the same sample size across groups. Skin wound tissue samples were collected for RNA extraction using the RNeasy Lipid Mini Kit (#74804, Qiagen, Hilden, Germany), in accordance with the manufacturer’s instructions. A total of 1 μg of RNA was then reverse transcribed into complementary DNA (cDNA) via the iScript cDNA Synthesis Kit (#1708891, Bio-Rad, Hercules, CA, USA). The resulting cDNA was then subjected to quantitative real-time PCR (qPCR) using the iQ™ SYBR Green Supermix (#1708880, Bio-Rad, Hercules, CA, USA) and a CFX96 Real-Time PCR Detection System (Bio-Rad, Hercules, CA, USA). Gene expression was normalized to the expression of the PPIA gene for each sample, and the relative fold change in gene expression was determined using the −ΔΔCt method. The primers employed for amplification were synthesized by Eurofins (Luxembourg) and are detailed in Table S2.

### 2.17. Quantification and Statistical Analysis

Images were analyzed using ImageJ software, whereas blood perfusion was analyzed using moorFLPI-2 Review Software V6.0. Data are presented as mean ± SEM or SD, as indicated in the corresponding figure legends. Normality was assessed using the Shapiro–Wilk test. For in vitro experiments, normally distributed data were analyzed using one-way ANOVA followed by Dunnett’s or Šídák’s multiple comparisons test, as appropriate. Non-normally distributed data were analyzed using the Kruskal–Wallis test followed by Dunn’s multiple comparisons test.

For in vivo experiments, normally distributed endpoint data were analyzed using one-way ANOVA followed by Šídák’s multiple comparisons test. Ordinal histological scores were analyzed using the Kruskal–Wallis test followed by Dunn’s multiple comparisons test. Longitudinal wound closure and blood perfusion data were analyzed separately for each genotype using two-way repeated-measures ANOVA, with Treatment and Day as factors, followed by Šídák’s multiple comparisons test. Šídák’s multiple comparisons test was used for predefined comparisons within each genotype, as appropriate for each experiment.

Potential outliers were assessed using ROUT (Q = 1%) and Grubbs’ tests. No outliers were identified or excluded. Missing values were not imputed. All statistical analyses were conducted using GraphPad Prism version 9 (GraphPad, San Diego, CA, USA).

## 3. Results

### 3.1. VCE-005.1 Protects Endothelial Cells Under Metabolic and Oxidative Stress

Endothelial dysfunction is a hallmark of diabetic wound healing, where hyperglycemia and oxidative stress impair endothelial survival, migration, and regenerative capacity [1,4,5]. We therefore first investigated whether VCE-005.1 protects endothelial cells under conditions that mimic the metabolic and oxidative stress associated with diabetes.

EA.hy926 endothelial cells were exposed to 30 or 80 mM glucose for four days to reproduce moderate and severe hyperglycemic stress, respectively, followed by treatment with increasing concentrations of VCE-005.1 for 24 h. Hyperglycemic conditions significantly reduced endothelial cell viability, whereas VCE-005.1 dose-dependently restored cell survival at both glucose concentrations (Figure 1A).

The effects of VCE-005.1 on endothelial repair were subsequently evaluated using an in vitro scratch assay. Exposure to both glucose concentrations markedly impaired endothelial migration and delayed wound closure, while treatment with VCE-005.1 significantly improved wound healing under both hyperglycemic conditions (Figure 1B and Figure S1).

Because oxidative stress is a major driver of endothelial senescence in diabetic wounds [9,10,11,12], HMEC-1 cells were exposed to H_2_O_2_, and senescence was assessed by SA-β-gal staining. Oxidative stress markedly increased the number of senescent cells, whereas VCE-005.1 significantly reduced SA-β-gal-positive cells (Figure 1C). These changes were accompanied by restoration of SIRT1 expression and downregulation of the senescence marker p21 (Figure 1D).

Collectively, these results demonstrate that VCE-005.1 protects endothelial cells against both metabolic and oxidative stress, preserving endothelial viability, migratory capacity, and resistance to stress-induced senescence, all of which are critical processes for effective vascular repair and wound healing [9,10,11,12,13,14,15].

### 3.2. VCE-005.1 Engages a B55α-Associated AMPK/SIRT1/HIF-1α Signaling Network

Having established the endothelial protective effects of VCE-005.1, we next investigated the molecular mechanisms underlying these responses. Previous studies demonstrated that VCE-005.1 stabilizes HIF-1α through a PP2A/B55α-dependent mechanism [18,22]. Here, we examined whether activation of the AMPK/SIRT1 signaling axis contributes to this response.

VCE-005.1 markedly increased AMPK phosphorylation and SIRT1 protein expression in endothelial cells. Pharmacological inhibition of AMPK with dorsomorphin (DS) markedly attenuated both responses, indicating that AMPK activity contributes to the upregulation of SIRT1 induced by VCE-005.1 (Figure 2A and Figure S2A).

To determine whether B55α participates in this signaling network, endothelial cells were transfected with B55α-specific siRNA prior to VCE-005.1 treatment. Efficient B55α knockdown reduced basal pAMPK and HIF-1α levels. VCE-005.1 increased both proteins in B55α-silenced cells; however, the response remained attenuated compared with control siRNA-transfected cells, indicating that B55α contributes to the activation of the AMPK/HIF-1α pathway by VCE-005.1 (Figure 2B and Figure S2B).

To further define the relationship between SIRT1 and HIF-1α, endothelial cells were transfected with SIRT1-specific siRNA before treatment with VCE-005.1. The silencing of SIRT1 markedly reduced HIF-1α induction by VCE-005.1, supporting the involvement of SIRT1 in VCE-005.1-induced HIF-1α regulation (Figure 2C and Figure S2C). Because AMPK and SIRT1 are functionally interconnected through cellular energy metabolism [31,32,33], we next evaluated SIRT1 enzymatic activity and the intracellular NAD^+^/NADH ratio. VCE-005.1 significantly increased SIRT1 activity (Figure 2D) together with the NAD^+^/NADH ratio (Figure 2E), consistent with activation of a metabolically favourable signaling program. At the highest concentration tested, the increase in the NAD^+^/NADH ratio was less pronounced, suggesting possible alterations in cellular redox homeostasis under these conditions.

Finally, the functional relevance of this signaling network was assessed using NIH-3T3-EPO-Luc reporter cells as a surrogate readout of HIF transcriptional activity. VCE-005.1 markedly increased EPO-driven luciferase activity, whereas pharmacological inhibition of AMPK (DS) or SIRT1 (EX527) significantly attenuated this response (Figure 2F). Furthermore, B55α silencing significantly attenuated VCE-005.1-induced phosphorylation of eNOS at Ser1177, supporting the functional integration of B55α within the endothelial signaling network activated by VCE-005.1 (Figure S3). Taken together, these data support the involvement of a B55α-associated AMPK/SIRT1/HIF-1α signaling network in the endothelial protective and pro-regenerative effects of VCE-005.1.

### 3.3. Development and Characterization of a VCE-005.1-Loaded Biologically Active PCL-Based Scaffold

To develop a local drug delivery system suitable for chronic wound treatment, VCE-005.1 was incorporated into 3D-printed polycaprolactone (PCL) scaffolds. The resulting scaffold exhibited a homogeneous architecture comparable to that of unloaded PCL-based scaffolds, with no evident alterations in scaffold morphology following drug incorporation (Figure 3A). FTIR spectroscopy was used to evaluate whether there were any interactions between PCL and VCE-005.1 (Figure 3B). Peaks at 1722 and 1720 cm^−1^ corresponding to C–O stretching bands were observed. This spectral region in PCL-based materials is typically attributed to overlapping bands characteristic of the PCL chain bonds [34]. In addition, the characteristic IR bands of PCL were identified, particularly at 1170 cm^−1^, which encompasses the peaks corresponding to OCO, COC, C–O, and C–C stretching vibrations. The FTIR spectrum of VCE-005.1 showed the characteristic absorption bands associated with triterpenoid hydroxamate derivatives. A broad band at 3260 cm^−1^ was due to O–H and N–H stretching vibrations, characteristic of compounds containing hydroxamate and hydroxyl groups. A peak at 3068 cm^−1^ associated with C=C stretching was also found [35]. Furthermore, the intense bands observed at 2930 and 2870 cm^−1^ corresponded to the asymmetric and symmetric stretching vibrations of the aliphatic CH_2_ groups, respectively, consistent with previous reports on triterpenoid structures [36]. The absorption band detected at 1641 cm^−1^ was assigned to the C=C stretching vibrations of the exocyclic isopropenyl group, characteristic of betulinic acid derivatives, while the band at 1730 cm^−1^ was associated with the C=O stretching vibrations corresponding to the hydroxamate carbonyl group [37]. No significant additional absorption bands or peak shifts were detected in the spectrum of the PCL + VCE-005.1 scaffold compared to that of the pure PCL scaffolds. This behavior is likely related to the low concentration of VCE-005.1 and the overlap between the compound’s characteristic absorption bands and those of the PCL matrix. However, the preservation of the characteristic PCL bands indicates that the incorporation of VCE-005.1 did not significantly alter the chemical structure of the polymeric scaffold. Furthermore, the biological activity observed for the VCE-005.1-loaded scaffolds indicates that VCE-005.1 retained its biological activity following incorporation into the PCL matrix. These results are similar to those obtained by Mathew et al., 2019 [38].

The incorporation of VCE-005.1 did not significantly modify the physicochemical properties of the scaffold. Contact angle measurements demonstrated comparable wettability between unloaded and drug-loaded scaffolds (Figure 3C), whereas compression testing showed similar mechanical behavior and compressive modulus for both formulations (Figure 3D), indicating that drug loading did not compromise scaffold integrity.

To determine whether VCE-005.1 remained biologically active after scaffold fabrication, conditioned media collected from the scaffolds over the experimental period were evaluated using. Drug-loaded scaffolds induced EPO-luciferase activity throughout the experimental period, providing functional evidence of the release of biologically active VCE-005.1 (Figure 3E). The 5 µM concentration of free VCE-005.1 used as a reference was selected based on its previously reported EC50 of 4.8 µM for HIF pathway activation in the EPO-Luc assay [28], thus providing a pharmacologically relevant reference for comparing the biological activity of VCE-005.1 released from the scaffolds. Since HIF activation by VCE-005.1 is critically dependent on the position of the hydroxamate moiety within the triterpenoid structure [28], preservation of EPO-Luc activity also provides indirect functional evidence that the pharmacologically relevant structure of VCE-005.1 was retained following scaffold fabrication.

Cell adhesion to the scaffold was subsequently assessed using FIBSV40-GFP fibroblasts. Cells readily attached to both unloaded and VCE-005.1-loaded scaffolds throughout the experimental period, with no evidence of reduced cell attachment following drug incorporation (Figure 3F). These findings indicate that VCE-005.1-loaded scaffolds promote fibroblast adhesion compared with control scaffolds and that VCE-005.1 retains its biological activity following incorporation into the PCL matrix.

### 3.4. Topical and Scaffold-Mediated Delivery of VCE-005.1 Accelerates Wound Healing

To evaluate the therapeutic efficacy of VCE-005.1 in vivo, two independent wound-healing studies were performed using diabetic db/db mice and normoglycemic db/+ controls. In the first study, VCE-005.1 was administered topically once daily, whereas in the second study, a single VCE-005.1-loaded PCL scaffold was applied to the wound at the beginning of the experiment and remained in place throughout the healing period.

Topical administration of VCE-005.1 progressively accelerated wound closure in both db/+ and db/db mice compared with their corresponding vehicle-treated groups (Figure 4A,C). The effect was particularly evident in diabetic animals, in which VCE-005.1 partially corrected the delayed healing response characteristic of the db/db phenotype. Importantly, the treatment also enhanced wound closure in normoglycemic db/+ mice, indicating that its pro-regenerative activity was not restricted to the diabetic condition. A significant Treatment × Day interaction was observed in db/+ mice (*p* = 0.046), whereas no significant interaction was detected in db/db mice.

A similar therapeutic response was observed following scaffold-mediated delivery. A single application of the VCE-005.1-loaded PCL scaffold significantly improved wound closure in both db/+ and db/db mice compared with the corresponding control scaffolds (Figure 4B,D). No significant treatment × day interaction was detected in either genotype. Thus, incorporation of VCE-005.1 into the PCL scaffold preserved its biological activity and enabled local treatment without repeated administration.

Because vascular insufficiency is a major determinant of impaired diabetic wound healing, local blood flow was additionally assessed in the scaffold study. Representative laser speckle images showed increased perfusion in wounds treated with the VCE-005.1-loaded scaffold (Figure 4E), and quantitative analysis confirmed a significant improvement in relative blood flow, particularly in diabetic mice (Figure 4F). A significant treatment × day interaction was observed in db/+ mice (*p* = 0.040), whereas the interaction did not reach statistical significance in db/db mice (*p* = 0.061).

Overall, these results demonstrate that VCE-005.1 accelerates wound healing following either repeated topical administration or a single scaffold-mediated application, supporting the PCL scaffold as an effective platform for local delivery.

### 3.5. Histological Evaluation of Wound Repair Following Topical and Scaffold-Mediated VCE-005.1 Treatment

To determine whether the accelerated wound closure induced by VCE-005.1 was accompanied by improved tissue regeneration, histological analyses were performed on wound sections collected at the end of the treatment period.

Representative hematoxylin and eosin (H&E)-stained sections demonstrated that topical administration of VCE-005.1 markedly improved the overall architecture of the regenerating tissue compared with vehicle-treated animals (Figure 5A). Histomorphometric analysis confirmed a significant increase in re-epithelialization and epidermal thickness index (ETI), together with enhanced collagen remodeling and a higher density of newly formed hair follicles, indicating a more advanced stage of tissue regeneration (Figure 5B). These effects were particularly evident in diabetic db/db mice, in which delayed wound healing was substantially improved.

Comparable histological improvements were observed following scaffold-mediated delivery of VCE-005.1. Representative H&E sections showed better restoration of tissue architecture in wounds treated with the VCE-005.1-loaded PCL scaffold than in those receiving unloaded scaffolds (Figure 5C). Quantitative analysis further demonstrated significant improvements in re-epithelialization, epidermal thickness index, collagen remodeling, and hair follicle density following scaffold-mediated treatment (Figure 5D), confirming that sustained local delivery effectively promotes tissue regeneration.

Overall, these findings demonstrate that both repeated topical administration and a single application of the VCE-005.1-loaded scaffold not only accelerate wound closure but also promote a more complete and structurally organized tissue repair response, providing the histological basis for the vascular remodeling described in the following sections.

### 3.6. Topical and Scaffold-Mediated Delivery of VCE-005.1 Promotes Vascular Regeneration

Having established that both treatment strategies improve the quality of tissue repair, we next investigated whether these histological changes were accompanied by enhanced vascular regeneration. Topical administration of VCE-005.1 markedly increased vascularization within the wound bed. Double immunofluorescence analysis demonstrated a significant increase in both mature blood vessels (CD31^+^/αSMA^+^) and endothelial progenitor-associated structures (CD31^+^/CD34^+^) compared with vehicle-treated animals (Figure 6A). These findings indicate that VCE-005.1 not only promotes neovascularization but also enhances vascular maturation during wound repair.

To determine whether the endothelial protective signaling pathway identified in vitro was also activated in vivo, we next evaluated the endothelial expression of B55α and SIRT1. Topical VCE-005.1 markedly increased the expression of both proteins within CD31^+^ endothelial cells (Figure 6B), supporting activation of the B55α/SIRT1-associated endothelial protective program during wound healing. These observations were further supported by increased expression of angiogenesis- and endothelial repair-related genes, including *Hgf*, *Epo*, *Vegf-A*, *Sirt1* and *B55α* (Figure S4A,B), together with enhanced endothelial Caveolin-1 expression (Figure S5A–C).

Importantly, scaffold-mediated delivery was also associated with vascular regenerative effects similar to those observed following topical administration. A single application of the VCE-005.1-loaded PCL scaffold significantly increased vessel density, vascular area, and the number of mature CD31^+^/αSMA^+^ vessels compared with unloaded scaffolds (Figure 7A,B). In parallel, scaffold treatment enhanced the expression of VEGF-A, Endoglin and HIF-1α within the regenerating tissue (Figure 7A,B). These findings were accompanied by increased expression of *Hgf*, *Epo*, *Vegf-A*, and *Endoglin* (Figure S4C), supporting sustained activation of angiogenic pathways throughout the healing process. Moreover, endothelial expression of Caveolin-1, B55α, and SIRT1 was also significantly increased in scaffold-treated wounds (Figures S5D–F and S6), demonstrating that scaffold-mediated delivery preserves the endothelial protective phenotype induced by topical VCE-005.1.

Taken together, these findings demonstrate that VCE-005.1 promotes vascular regeneration through coordinated enhancement of endothelial activation, vessel maturation, and angiogenic signaling. Importantly, the VCE-005.1-loaded PCL scaffold produced a similar pattern of biological effects following a single application, highlighting its potential as an effective local drug delivery strategy for diabetic wound repair.

### 3.7. Topical and Scaffold-Mediated Delivery of VCE-005.1 Attenuates Inflammatory Responses During Wound Healing

Because persistent inflammation is a hallmark of chronic diabetic wounds and contributes to impaired tissue repair [39], we finally evaluated whether VCE-005.1 modulates inflammatory cell infiltration within the wound bed.

Topical administration of VCE-005.1 significantly reduced the accumulation of F4/80-positive macrophages and MPO-positive neutrophils compared with vehicle-treated animals (Figure 8A,B). A similar reduction in inflammatory cell infiltration was observed following scaffold-mediated delivery, indicating that sustained local release of VCE-005.1 effectively attenuates the inflammatory response associated with diabetic wound healing (Figure 8C,D).

To further characterize the inflammatory milieu, the expression of genes associated with pro-inflammatory (M1) and reparative/pro-angiogenic (M2) responses was evaluated. Scaffold-mediated delivery of VCE-005.1 significantly reduced the expression of the pro-inflammatory mediators *Il6*, *Il1β* and *Il12*, while increasing the expression of the anti-inflammatory cytokine *Il10* (Figure S7), supporting the establishment of a tissue environment that favors resolution of inflammation and tissue repair.

## 4. Discussion

Diabetic foot ulcers remain one of the most challenging complications of diabetes because impaired endothelial function, persistent inflammation and defective vascular regeneration prevent efficient tissue repair. Although several pro-angiogenic strategies, including growth factor- and cell-based therapies, have shown encouraging preclinical results, their clinical translation has been limited by modest efficacy, poor persistence at the wound site and safety concerns [1,2,3,4,5,40]. Consequently, there is considerable interest in pharmacological approaches capable of restoring endogenous vascular repair mechanisms while preserving tissue homeostasis.

The present study demonstrates that VCE-005.1 promotes diabetic wound healing through a coordinated mechanism involving endothelial protection, vascular regeneration and resolution of inflammation. Beyond confirming the therapeutic efficacy of topical VCE-005.1, we demonstrate that these biological effects can be reproduced by a single application of a VCE-005.1-loaded PCL scaffold, highlighting the translational potential of scaffold-mediated local drug delivery for chronic diabetic wounds.

Previous studies from our group identified VCE-005.1 as a hypoxia-mimetic betulinic acid hydroxamate that stabilizes HIF-1α through PP2A/B55α-associated signaling and specific post-translational inhibition of PHD2 [18,22]. Here, we extend these observations by demonstrating that VCE-005.1 engages a broader B55α-associated signaling network involving AMPK, SIRT1 and HIF-1α. Consistent with the well-established interplay between AMPK and SIRT1 [31,32,33], VCE-005.1 increased AMPK phosphorylation, SIRT1 expression and activity, elevated the NAD^+^/NADH ratio and enhanced HIF-dependent transcriptional activity. Although the precise molecular hierarchy remains to be established, our findings support a coordinated signaling network that promotes endothelial adaptation to metabolic and hypoxic stress.

The functional relevance of this signaling network became evident under conditions that reproduce the diabetic wound microenvironment. Chronic hyperglycemia and oxidative stress impair endothelial function and angiogenesis [9,10,11,12,13,14,15], whereas VCE-005.1 preserved endothelial viability and migration, reduced senescence, restored SIRT1 expression and increased eNOS phosphorylation, supporting maintenance of endothelial homeostasis under diabetic conditions. These endothelial protective effects translated into a robust regenerative response in vivo. Topical VCE-005.1 accelerated wound closure while improving the quality of tissue repair, as reflected by enhanced re-epithelialization, collagen remodeling and hair follicle regeneration in both diabetic and normoglycemic animals. Improved tissue architecture was accompanied by increased vascular maturation and neovascularization, evidenced by a higher abundance of CD31^+^/αSMA^+^ mature vessels and CD31^+^/CD34^+^ endothelial structures, together with enhanced endothelial expression of B55α, SIRT1 and Caveolin-1. Consistent with these findings, HIF-1α upregulation was associated with increased expression of its downstream target genes, including *Vegf-A*, *Hgf* and *Epo*, supporting activation of a coordinated hypoxia-responsive program that promotes vascular regeneration and tissue repair.

Interestingly, VCE-005.1 also promoted wound healing in normoglycemic db/+ mice, suggesting that activation of the B55α-associated signaling network enhances physiological tissue repair independently of diabetes. This observation raises the possibility that VCE-005.1 may also be beneficial in other chronic wounds associated with impaired vascular repair.

In parallel with its vascular effects, VCE-005.1 promoted resolution of inflammation during wound healing. Both topical and scaffold-mediated delivery reduced macrophage and neutrophil infiltration, while the scaffold formulation decreased the expression of *Il6, IL-1β*, and *Il12* and increased *Il10*. Together, these findings indicate that VCE-005.1 facilitates the transition toward a tissue environment that supports vascular regeneration and wound repair.

Local drug delivery systems capable of providing sustained release while minimizing repeated administration are particularly attractive for chronic wound treatment [23,24,25,26,27]. Incorporation of VCE-005.1 into a PCL scaffold preserved both its physicochemical properties and biological activity, while EPO-luciferase reporter activity provided functional evidence of the release of biologically active VCE-005.1, and fibroblast cell adhesion was significantly increased on VCE-005.1-loaded scaffolds compared with control scaffolds. However, this functional assay does not provide quantitative release kinetics or direct analytical confirmation of drug recovery and chemical stability. These parameters will require dedicated analytical characterization during further pharmaceutical development.

PCL provides mechanical stability and slow degradation but, due to its hydrophobic nature, has lower fluid absorption than hydrogel-based systems such as GelMA. Although the 3D-printed scaffold supported sustained cell adhesion, porosity and cell migration were not quantitatively evaluated and will require further characterization. In vivo, the scaffold remained at the wound during active healing and subsequently detached spontaneously as tissue repair progressed, without apparent interference with wound closure.

From a translational perspective, the most relevant finding of this study is that a single application of the VCE-005.1-loaded scaffold produced a qualitatively similar pattern of biological responses to that observed with repeated topical administration, including accelerated wound closure, improved tissue remodeling, enhanced vascular regeneration, increased perfusion, and reduced inflammatory cell infiltration. These findings highlight scaffold-mediated local delivery of VCE-005.1 as a practical therapeutic strategy for chronic diabetic wounds.

The present study has several limitations that should be considered in the context of further development. The splinted excisional wound model in db/db mice reproduces impaired diabetic cutaneous wound healing but does not encompass the full clinical complexity of human DFU, including plantar biomechanics, neuropathy, peripheral arterial disease, and infection. In addition, VCE-005.1 release from the scaffold was evaluated through biological activity rather than by direct analytical quantification. Future studies should therefore establish the quantitative release profile and local pharmacokinetics of VCE-005.1, including drug concentrations in wound tissue and plasma over time, and validate its therapeutic activity in more advanced preclinical models. Furthermore, scaffold compatibility was assessed using fibroblast adhesion, and further viability and cytotoxicity studies in endothelial cells will be required for preclinical development. Nevertheless, the consistent biological responses observed across in vitro studies, topical administration, and scaffold-mediated delivery provide strong support for the continued preclinical development of VCE-005.1.

In conclusion, our findings identify VCE-005.1 as a promising candidate for the treatment of diabetic foot ulcers. By simultaneously preserving endothelial function, promoting vascular regeneration, and facilitating the resolution of inflammation, VCE-005.1 addresses several of the key pathological processes that impair diabetic wound healing. Coupled with the successful development of a biologically active VCE-005.1-loaded PCL scaffold, these findings provide a strong rationale for the further translational development of VCE-005.1 for diabetic foot ulcers and other chronic wounds associated with vascular insufficiency.

## Figures and Tables

**Figure 1:**
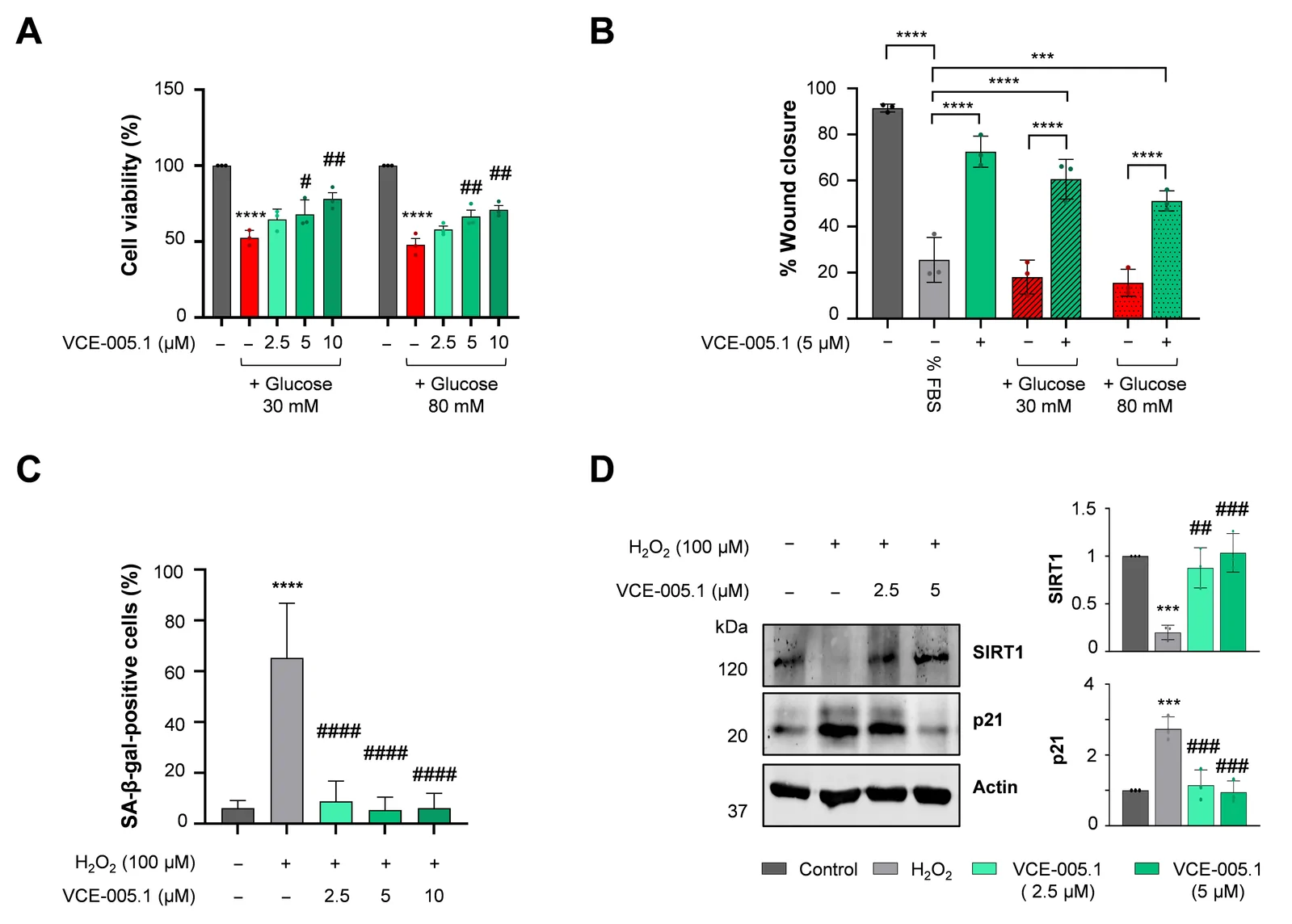
VCE-005.1 protects endothelial cells under metabolic and oxidative stress. (**A**) EA.hy926 cells were treated with VCE-005.1 under high glucose conditions, and cell viability was assessed by MTT assay. Data are presented as mean ± SD (n = 3). (**B**) In vitro wound healing assay in EA.hy926 cells exposed to high glucose (30 or 80 mM D-glucose) for 24 h and treated with VCE-005.1 (5 µM). Wound closure was quantified 24 h after scratching. Data are presented as mean ± SD (n = 3). (**C**) HMEC-1 cells were exposed to H_2_O_2_ (100 µM) in the presence or absence of VCE-005.1. Cellular senescence was assessed by SA-β-gal staining and quantified as the percentage of SA-β-gal-positive cells per field. Data are presented as mean ± SD (n = 3). (**D**) SIRT1 and p21 protein expression was analyzed by Western blot in HMEC-1 cells exposed to H_2_O_2_ (100 µM) in the presence or absence of VCE-005.1. Protein levels were normalized to β-actin. Representative blots and β-actin-normalized protein quantification are shown. Data are presented as mean ± SD (n = 3). Statistical analyses were performed using one-way ANOVA followed by Dunnett’s multiple comparisons test. Significance is indicated in the corresponding panels as *** *p* < 0.001, and **** *p* < 0.0001. For panel (**A**), **** *p* < 0.0001 vs. control, and # *p* < 0.05 and ## *p* < 0.01 vs. high glucose-treated cells. For panel (**B**), *** *p* < 0.001 and **** *p* < 0.0001 for the comparisons indicated by the brackets. For panels (**C**,**D**), *** *p* < 0.001 and **** *p* < 0.0001 vs. control; ## *p* < 0.01, ### *p* < 0.001, and #### *p* < 0.0001 vs. H_2_O_2_-treated cells.

**Figure 2:**
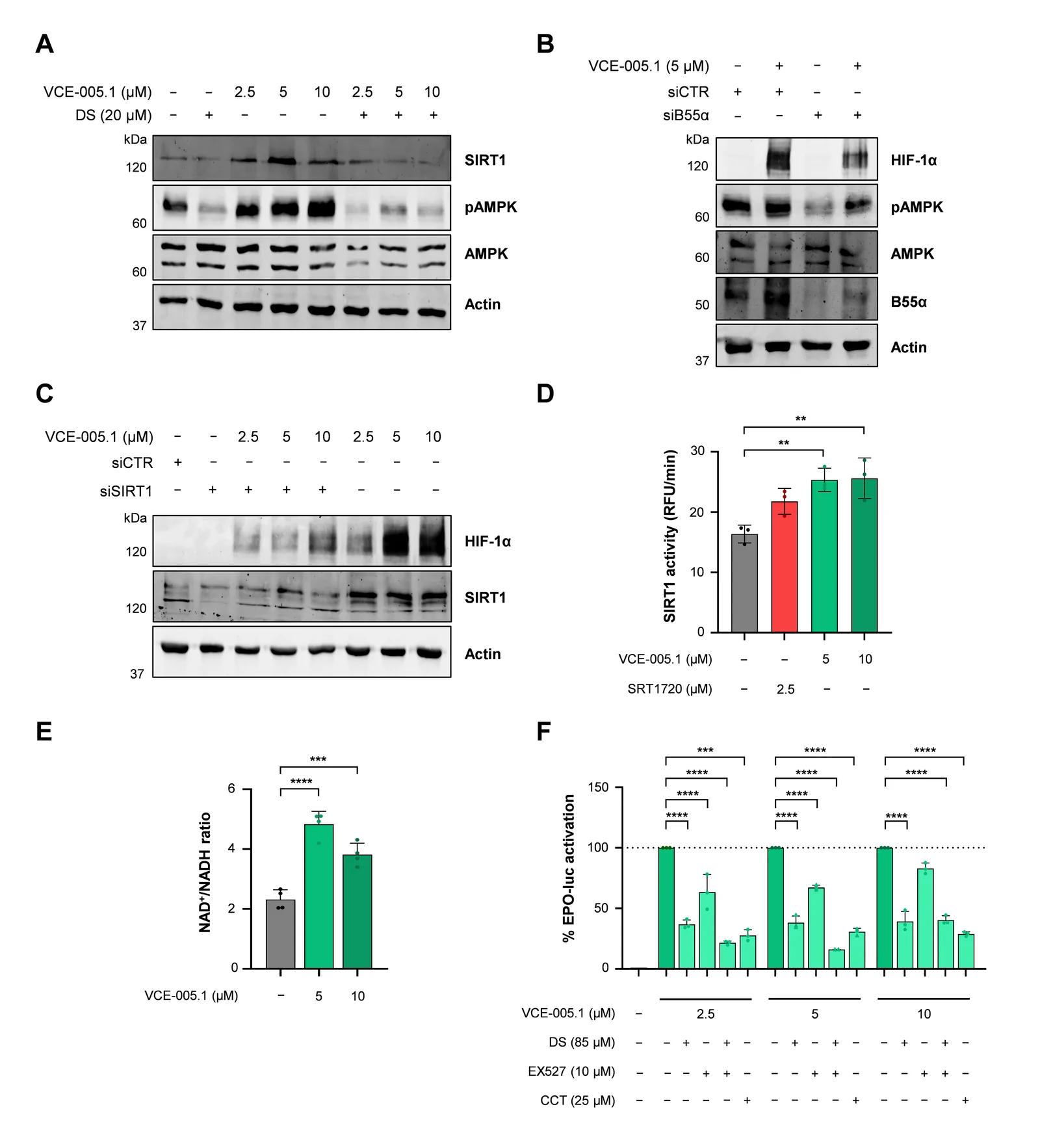
VCE-005.1 engages a B55α-associated AMPK/SIRT1/HIF-1α signaling network. (**A**) EA.hy926 cells were pre-incubated with or without DS for 30 min and subsequently treated with VCE-005.1 for 5 h (n = 3). (**B**) EA.hy926 cells were transfected with siB55α or scrambled siRNAs (siCTR) followed by stimulation with VCE-005.1 for 5 h (n = 3). (**C**) EA.hy926 cells were subjected to transfection with siSIRT1 or scrambled siRNAs (siCTR), followed by treatment with VCE-005.1 for 5 h (n = 3). (**D**) SIRT1 activity following VCE-005.1 treatment. SRT1720 was used as the positive control. Data represent the mean ± SD (n = 3). (**E**) NAD^+^/NADH ratio was measured using the EA.hy926 cell line was treated with different concentrations of VCE-005.1 for 3 h. Data represent the mean ± SD (n = 4). (**F**) In the presence or absence of AMPK, SIRT1, and HSP90 inhibitors, NIH-3T3-EPO-luc cells were treated with VCE-005.1 for 6 h. After this, luciferase activity was measured and expressed as a percentage of EPO-luc activation, considering the value of VCE-005.1 as 100% activation. Data represent the mean ± SD (n = 3). Statistical significance was determined using one-way ANOVA followed by Dunnett’s multiple comparisons test (panels (**D**,**E**)) or the Kruskal–Wallis test followed by Dunn’s multiple comparisons test (panel (**F**)). Statistical significance is indicated in the corresponding panels as ** *p* < 0.01, *** *p* < 0.001, and **** *p* < 0.0001.

**Figure 3:**
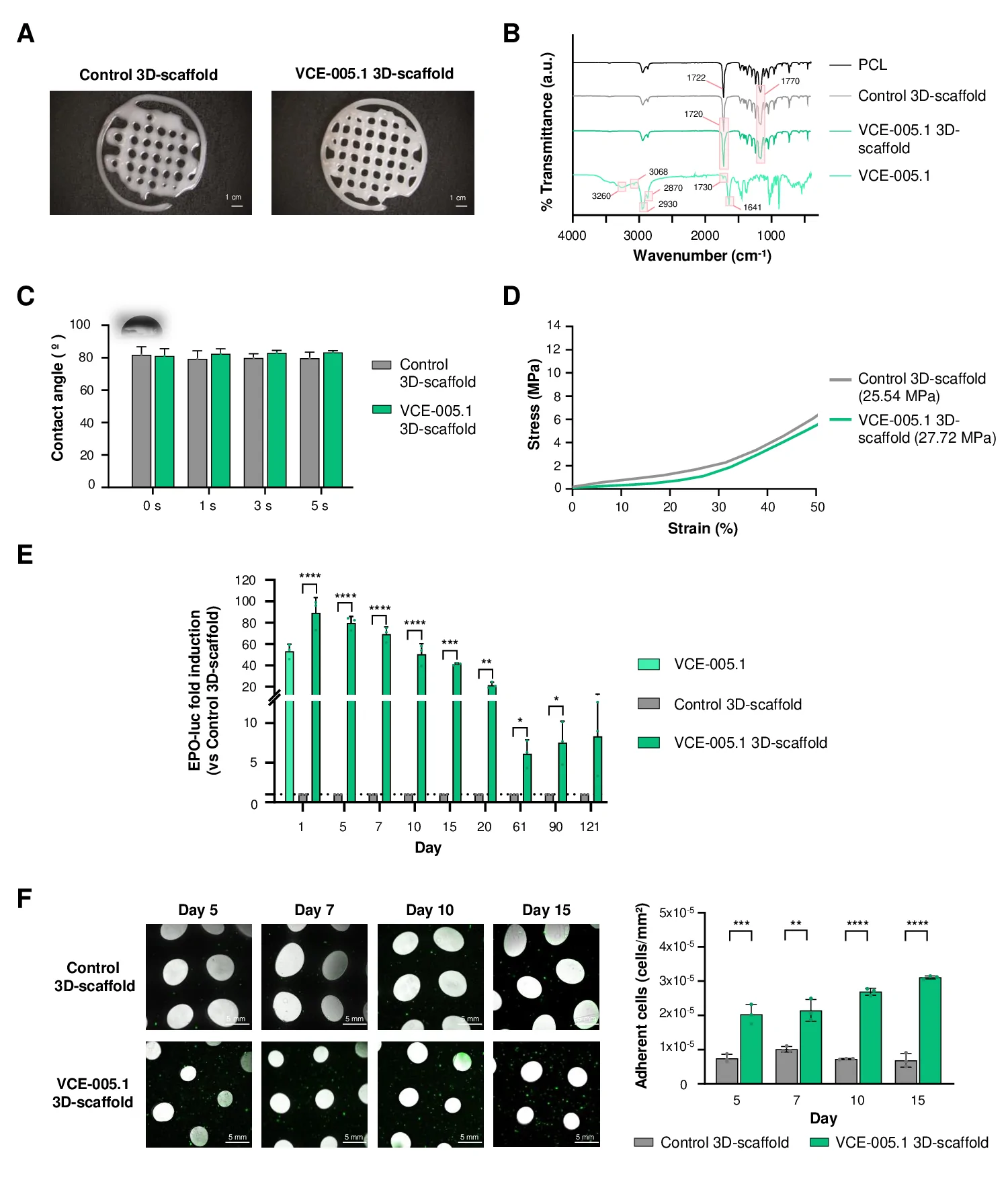
Development of a biologically active VCE-005.1-loaded PCL scaffold for local drug delivery. (**A**) Representative images of Control 3D-scaffold and VCE-005.1 3D-scaffold. (**B**) Fourier-transform infrared spectroscopy (FTIR) spectra of PCL, Control 3D-scaffold, VCE-005.1 3D-scaffold, and VCE-005.1. (**C**) Water contact angle measurements of control and VCE-005.1. 3D scaffolds at different time points. (**D**) Stress–strain curves and compressive modulus (MPa) of Control and VCE-005.1 3D scaffolds. (**E**) Induction of EPO-Luc reporter activity in NIH-3T3 cells following stimulation with VCE-005.1 (5 µM) or with supernatants collected from VCE-005.1 3D-scaffolds over time. Data are expressed as fold induction relative to the control 3D-scaffold and are presented as mean ± SD (n = 3). (**F**) Adhesion of FIBSV40-GFP fibroblasts on control and VCE-005.1 3D-scaffolds. Representative fluorescence and bright-field images acquired over a 15-day period at ×5 magnification are shown. Merged fluorescence images (green) indicate GFP-positive adherent cells. Quantification of adherent cells per scaffold area (cells/mm^2^) was performed at all-time points (n = 3). Statistical significance was determined using one-way ANOVA followed by Dunnett’s multiple comparisons test (panel (**E**)) or one-way ANOVA followed by Šídák’s multiple comparisons test (panel (**F**)). Statistical significance is indicated in the corresponding panels as * *p* < 0.05, ** *p* < 0.01, *** *p* < 0.001, and **** *p* < 0.0001.

**Figure 4:**
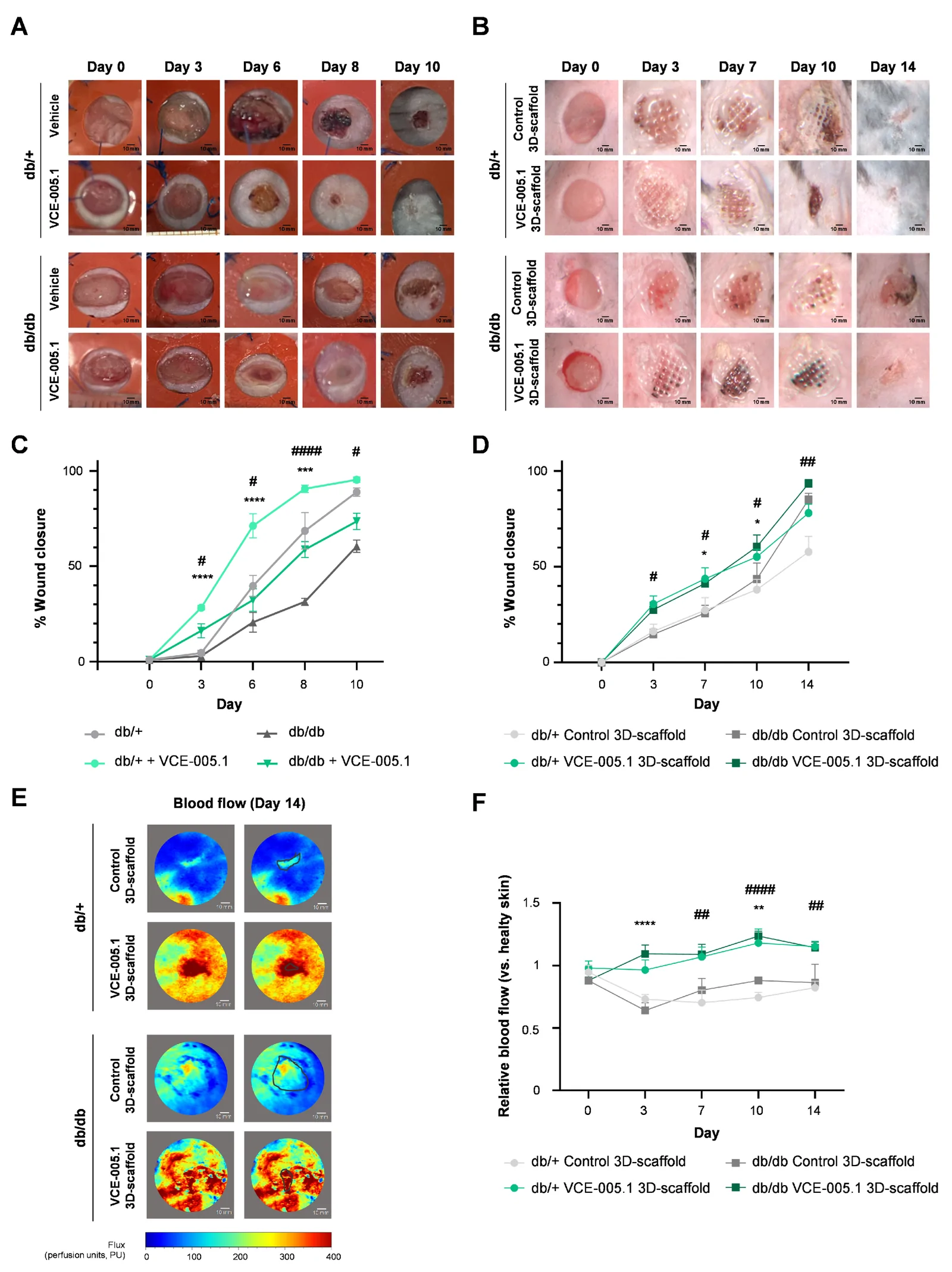
Therapeutic efficacy of topical and scaffold-mediated VCE-005.1 delivery in diabetic wound healing. (**A**) Representative images of wounds throughout the healing process in db/+ and db/db mice treated topically with vehicle or VCE-005.1. Scale bars = 10 mm. (**B**) Representative images of wound healing in db/+ and db/db mice treated with control or VCE-005.1-loaded 3D-scaffolds at days 0, 3, 7, 10, and 14. Scale bars = 10 mm. (**C**) Quantitative analysis of wound closure following topical treatment at days 0, 3, 6, 8, and 10 (*n* = 4 animals for vehicle-treated groups and n = 5 animals for VCE-005.1-treated groups). (**D**) Quantitative assessment of wound closure during the 14-day healing period in db/+ and db/db mice treated with control or VCE-005.1-loaded 3D-scaffolds (n = 5 animals for control scaffold groups and n = 6 animals for VCE-005.1-loaded scaffold groups). (**E**) Representative laser speckle contrast images of blood perfusion at day 14. For each group, the left image shows the perfusion map, while the right image shows the same map with the wound bed outlined in black. The wound bed was excluded from the analysis to determine perfusion in the surrounding periwound tissue. All perfusion maps are displayed using the same colour scale (0–400 perfusion units, PU). Scale bars = 10 mm. (**F**) Quantitative assessment of blood perfusion, normalized to healthy skin, over the 14-day experimental period (n = 5 animals for control scaffold groups and n = 6 animals for VCE-005.1-loaded scaffold groups). Data are presented as mean ± SEM. For panels (**C**,**D**,**F**), normality was assessed using the Shapiro–Wilk test. Longitudinal data were analyzed separately for each genotype using two-way repeated-measures ANOVA, with treatment and day as factors, followed by Šídák’s multiple comparisons test. Statistical significance is indicated in the corresponding panels as * *p* < 0.05, ** *p* < 0.01, *** *p* < 0.001 and **** *p* < 0.0001 for comparisons between db/+ and db/+ VCE-005.1 or between db/+ Control 3D-scaffolds and db/+ VCE-005.1-loaded 3D-scaffolds; # *p* < 0.05, ## *p* < 0.01 and #### *p* < 0.0001 for comparisons between db/db and db/db + VCE-005.1 or between db/db Control 3D-scaffolds and db/db VCE-005.1-loaded 3D-scaffolds.

**Figure 5:**
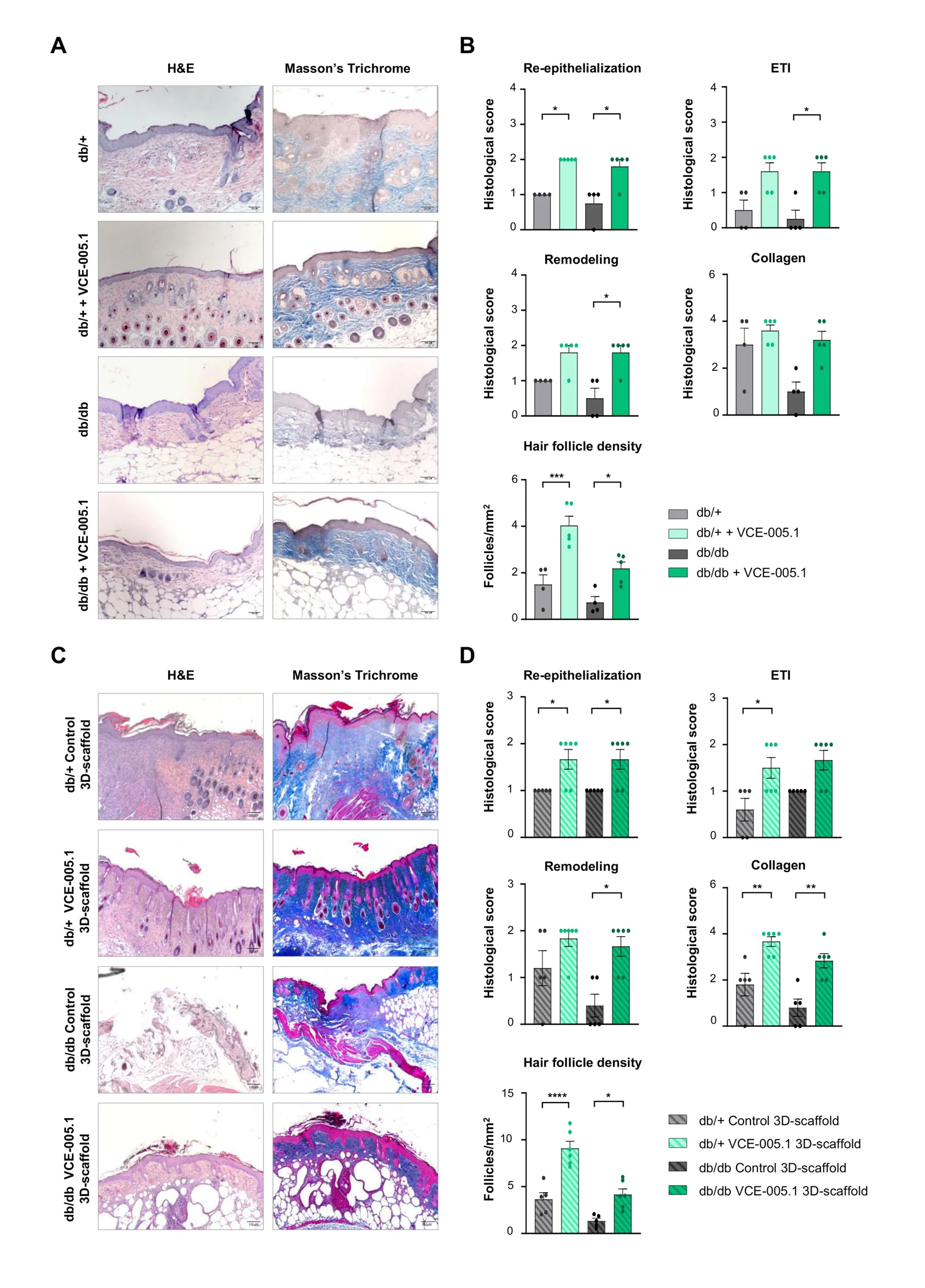
Histological evaluation of wound repair following topical and scaffold-mediated VCE-005.1 treatment. (**A**) Tissue samples were stained with H&E or Masson’s trichrome on day 10. Images were acquired at ×10 magnification. Scale bars = 100 μm. (**B**) Quantitative evaluation of wound healing parameters, including re-epithelialization, epidermal thickness index (ETI), tissue remodeling, collagen deposition, and hair follicle density, based on H&E and Masson’s trichrome staining from the topical treatment model (*n* = 4 animals for vehicle-treated groups and n = 5 animals for VCE-005.1-treated groups). (**C**) Representative histological images of wound tissues collected on day 14 following treatment with Control or VCE-005.1 3D-scaffolds and stained with H&E or Masson’s trichrome. Images were acquired at ×10 magnification. Scale bars = 100 μm. (**D**) Quantitative evaluation of wound healing parameters, including re-epithelialization, epidermal thickness index (ETI), tissue remodeling, collagen deposition, and hair follicle density, based on H&E and Masson’s trichrome staining from the 3D-scaffold treatment model (n = 5 animals for control scaffold groups and n = 6 animals for VCE-005.1-loaded scaffold groups). Histological scoring criteria were defined as follows: re-epithelialization (0: none (0%), 1: partial (1–95%), 2: complete (95–100%)); ETI (0: hypoplasia (<95%), 1: hypertrophy (>105%), 2: healed wound (95–105%)); tissue remodeling (0: not remodeled, 1: partial, 2: complete); collagen deposition (0: absent, 1: minimal granulation tissue, 2: mild granulation tissue, 3: moderate granulation tissue, 4: marked granulation tissue). Data are presented as mean ± SEM. Each data point represents one animal. For panels (**B**,**D**), re-epithelialization, ETI, tissue remodeling, and collagen deposition scores were analyzed using the Kruskal–Wallis test followed by Dunn’s multiple comparisons test. Hair follicle density (follicles/mm^2^) was assessed for normality using the Shapiro–Wilk test and analyzed using one-way ANOVA followed by Šídák’s multiple comparisons test. Statistical significance is indicated in the corresponding panels as * *p* < 0.05, ** *p* < 0.01, *** *p* < 0.001, and **** *p* < 0.0001.

**Figure 6:**
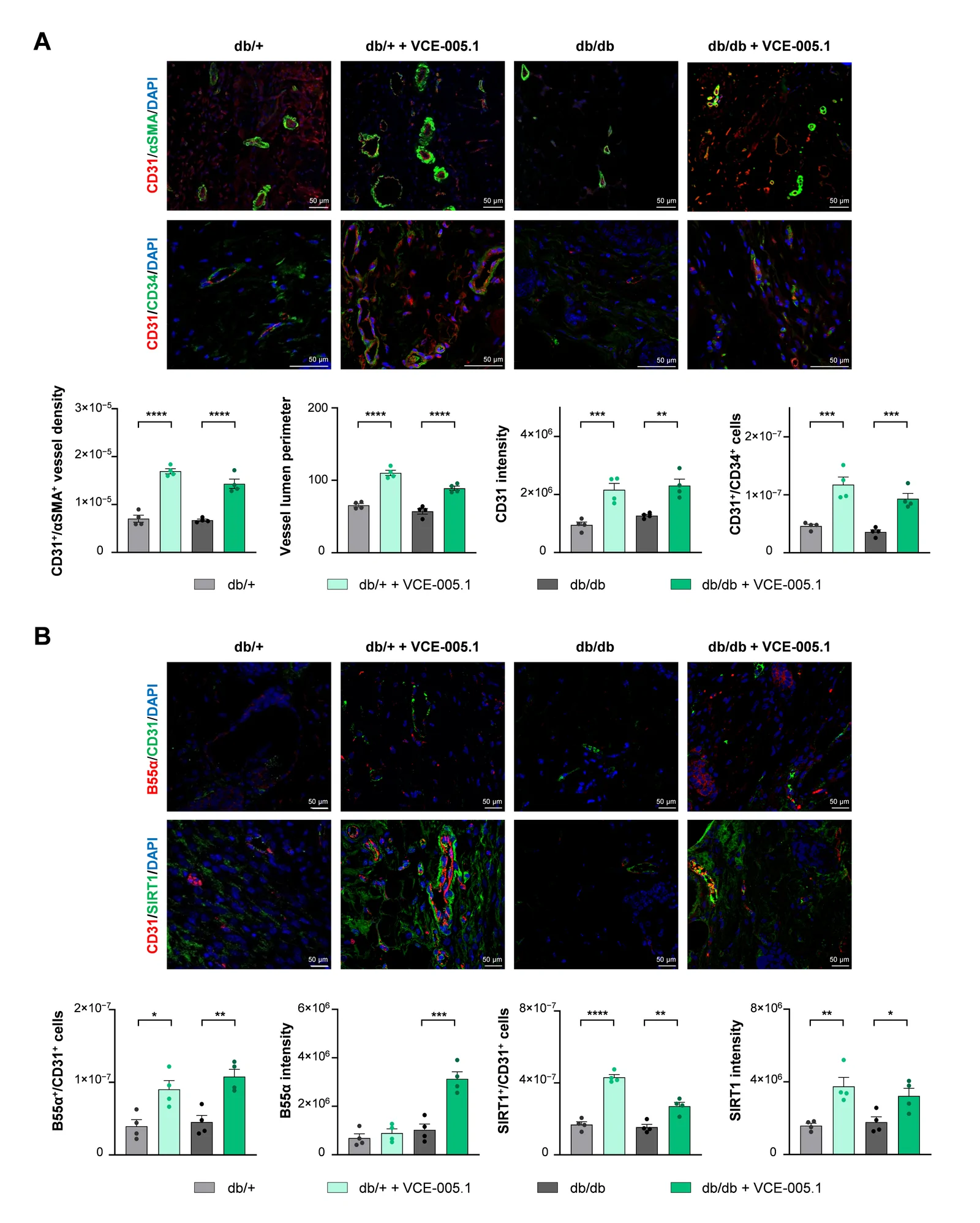
Topical VCE-005.1 treatment promotes vascular regeneration and restores endothelial SIRT1 expression in diabetic wounds. (**A**) Representative confocal double immunofluorescence staining of CD31 (red)/αSMA (green) and CD34 (green)/CD31 (red) in wound sections. CD31^+^/αSMA^+^ vessel density, vessel lumen perimeter, CD34 integrated fluorescence density, and CD31^+^/CD34^+^ cell density were quantified. Vessel and cell densities were calculated as the number of double-positive vessels or cells divided by the corresponding tissue area analyzed and expressed as vessels/μm^2^ or cells/μm^2^, respectively. CD31 was used as an endothelial marker, αSMA as a perivascular marker associated with vessel maturation, and CD34 as a marker of newly formed vessels. Images were acquired at ×40 magnification. Scale bars = 50 μm. (**B**) Representative confocal double immunofluorescence staining of B55α (red)/CD31 (green) and SIRT1 (green)/CD31 (red) in wound sections, together with their corresponding quantifications. B55α^+^/CD31^+^ and SIRT1^+^/CD31^+^ cell densities were calculated as the number of double-positive cells divided by the corresponding tissue area analyzed and expressed as cells/μm^2^. B55α and SIRT1 fluorescence was quantified as integrated fluorescence density. Images were acquired at ×40 magnification. Scale bars = 50 μm. Data are presented as mean ± SEM (n = 4 animals per group). Each data point represents one animal. Normality was assessed using the Shapiro–Wilk test. Statistical significance was determined using one-way ANOVA followed by Šídák’s multiple comparisons test. Statistical significance is indicated in the corresponding panels as * *p* < 0.05, ** *p* < 0.01, *** *p* < 0.001, and **** *p* < 0.0001.

**Figure 7:**
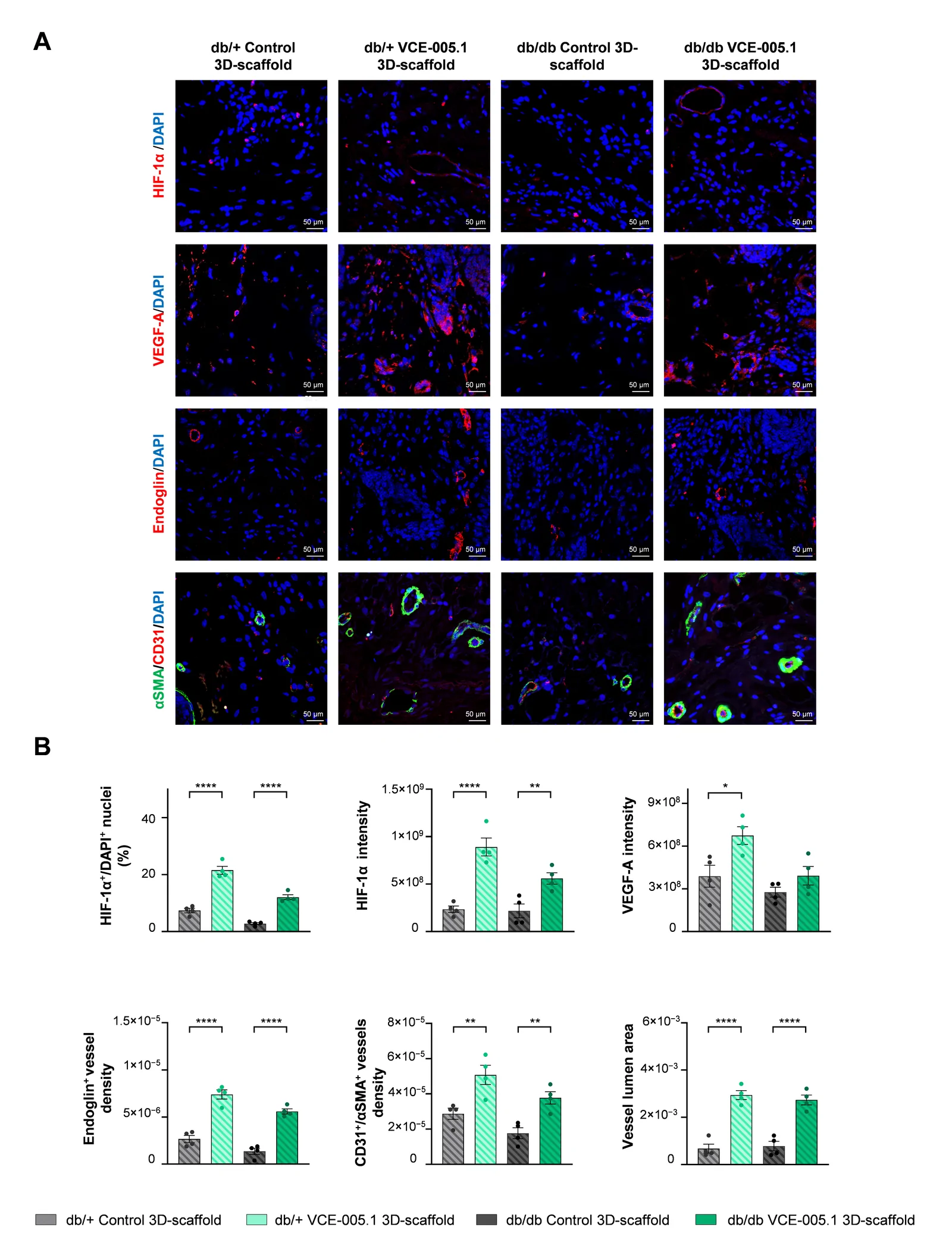
VCE-005.1 3D-scaffolds promote angiogenic and reparative responses during wound healing. (**A**) Representative confocal immunofluorescence staining of HIF-1α (red)/DAPI (blue), VEGF-A (red)/DAPI (blue), Endoglin (red)/DAPI (blue), and αSMA (green)/CD31 (red)/DAPI (blue) in wound tissue sections. HIF-1α was used as a marker of hypoxia-responsive signaling, VEGF-A as a pro-angiogenic factor, endoglin as a marker associated with endothelial activation and angiogenesis, CD31 as an endothelial marker, and αSMA as a perivascular cell marker associated with vessel maturation. Images were acquired at ×40 magnification. Scale bars = 50 μm. (**B**) Quantification of HIF-1α^+^/DAPI^+^ nuclei (%), HIF-1α and VEGF-A integrated fluorescence density, Endoglin^+^ and CD31^+^/α-SMA^+^ vessel density, calculated as the number of positive vessels divided by the corresponding tissue area analyzed (vessels/μm^2^), and vessel lumen area relative to the analyzed tissue area. Results are presented as mean ± SEM (n = 4 animals per group), and each data point represents one animal. Normality was assessed using the Shapiro–Wilk test. Statistical significance was determined using one-way ANOVA followed by Šídák’s multiple comparisons test. Statistical significance is indicated in the corresponding panels as * *p* < 0.05, ** *p* < 0.01, and **** *p* < 0.0001.

**Figure 8:**
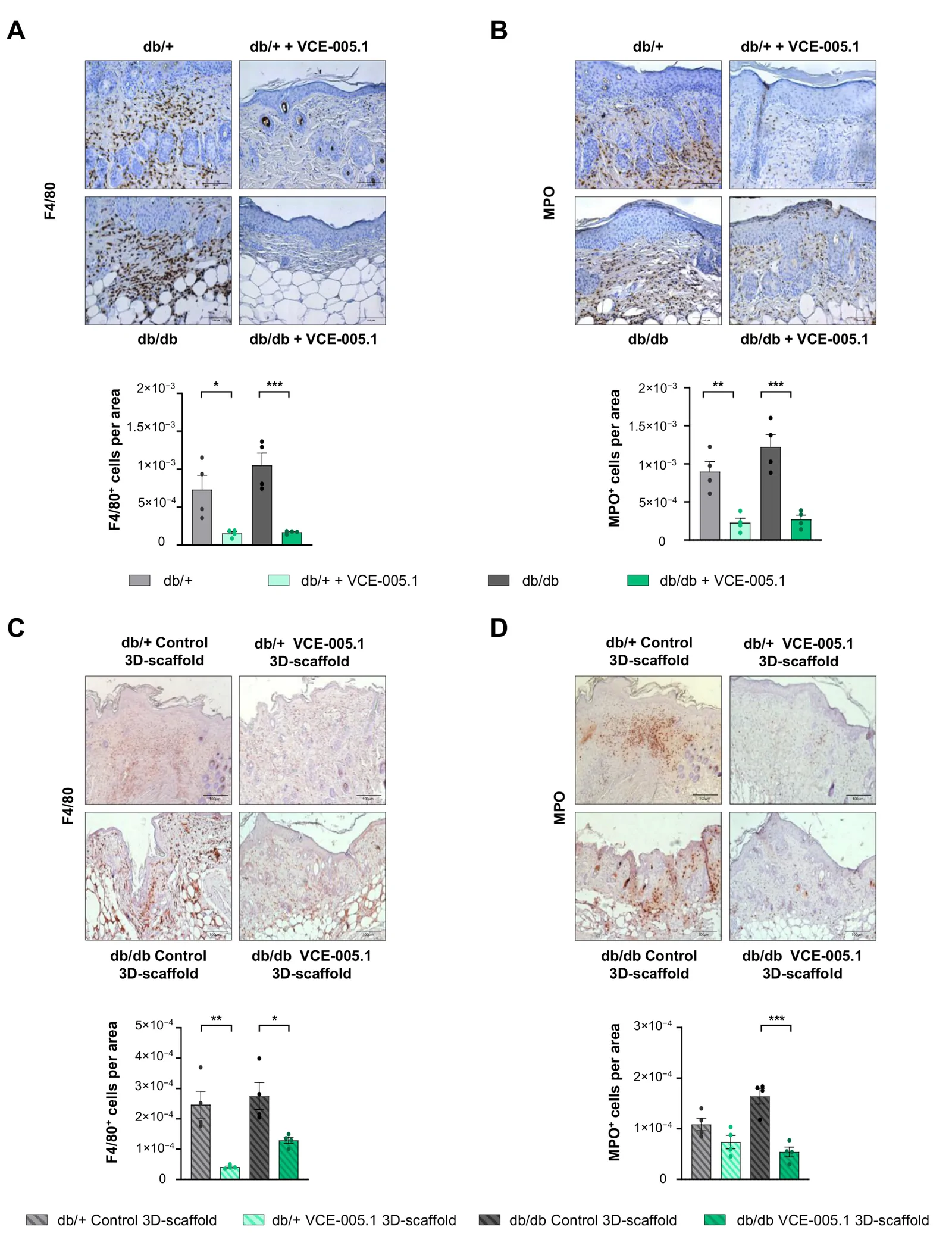
Topical and scaffold-mediated delivery of VCE-005.1 reduces inflammatory cell infiltration in diabetic wounds. (**A**) Representative immunohistochemical staining of F4/80 in wound tissue sections from db/+ and db/db mice treated with vehicle or topical VCE-005.1, together with the corresponding quantification of F4/80^+^ macrophage density (cells/µm^2^). (**B**) Immunohistochemical detection of myeloperoxidase (MPO) in the corresponding wound tissue sections, together with the quantification of MPO^+^ neutrophil density (cells/µm^2^). (**C**) Representative immunohistochemical staining of F4/80 in wound tissue sections from mice treated with control or VCE-005.1-loaded 3D-scaffolds, together with the corresponding quantification of F4/80^+^ macrophage density (cells/µm^2^). (**D**) Immunohistochemical detection of MPO in wound tissue sections from mice treated with control or VCE-005.1-loaded 3D-scaffolds, together with the quantification of MPO^+^ neutrophil density (cells/µm^2^). All images were acquired at ×20 magnification. Scale bars = 100 μm. Data are presented as mean ± SEM (n = 4 animals per group). Each dot represents one animal. Normality was assessed using the Shapiro–Wilk test. Statistical significance was determined using one-way ANOVA followed by Šídák’s multiple comparisons test. Statistical significance is indicated in the corresponding panels as * *p* < 0.05, ** *p*< 0.01, *** *p* < 0.001.

## Data Availability

The datasets used and/or analysed during the current study are available from the corresponding authors on reasonable request.

## Supplementary Materials

The following supporting information can be downloaded at https://www.mdpi.com/article/10.3390/pharmaceutics18101200/s1: Figure S1. Bright-field images of endothelial wound closure following VCE-005.1 treatment under basal and hyperglycemic conditions; Figure S2. Signaling pathways involved in VCE-005.1-induced HIF-1α activation; Figure S3. Effect of B55α silencing on VCE-005.1-induced eNOS phosphorylation; Figure S4. Gene expression of angiogenic and tissue repair markers in wound skin; Figure S5. VCE-005.1 induces CAV1 in vivo; Figure S6. VCE-005.1 3D-scaffolds enhance B55α and SIRT1 expression during wound healing; Figure S7. Effect of VCE-005.1-loaded 3D-scaffolds on inflammatory gene expression in wound tissue. Table S1. Antibodies Used for Experimental Procedures. Table S2. qRT-PCR Primers and Their Sequences.
