# Supplementary material for: Topical and Scaffold-Mediated Delivery of VCE-005.1 Enhances Diabetic Wound Healing Through Endothelial Protection and Vascular Regeneration
Source: Pharmaceutics. 2026 Sep 22;18(10):1200. doi: 10.3390/pharmaceutics18101200 (PMC13708403; doi:10.3390/pharmaceutics18101200)
Supplement: Supplementary file 1 [file pharmaceutics-18-01200-s001.zip › pharmaceutics-4491284-supplementary.pdf]

Supplementary Information

Topical and Scaffold-Mediated Delivery of VCE-005.1 Enhances Diabetic Wound Healing through Endothelial Protection and Vascular Regeneration

Isabel Lastres-Cubillo[1,2,3], María E. Prados[1,2,3], Esther Rincón[4], Eduardo Espinosa[4], Juan Domínguez-Robles[5], Ilyas Essadki-Aittaji[5], Adela García-Martín[1,2,3*], and Eduardo Muñoz[1,2,3*].

[1] Maimonides Biomedical Research Institute of Córdoba (IMIBIC), Córdoba, Spain.

[2] Hospital Universitario Reina Sofía, Córdoba, Spain.

[3] Cellular Biology, Physiology and Immunology Department, University of Córdoba, Spain.

[4] BioPrEn Group (RNM940), Chemical Engineering Department, Instituto Químico para Energía y el Medioambiente (IQUEMA), Faculty of Science, University of Córdoba, 14014, Córdoba, Spain.

[5] Department of Pharmacy and Pharmaceutical Technology, Faculty of Pharmacy, Universidad de Sevilla, 41012, Seville, Spain.

Supplementary Tables

Table S1. Antibodies Used for Experimental Procedures

Antibody	Dilution	Reference	Distributor
**HIF-1α**	1:1000<br>1:100	#ab179483	Abcam
**β-actin**	1:5000	#A5316	Merck
**B55α**	1:1000<br>1:250	#5689S<br>#4953S	Cell Signaling
**phospho-AMPK**	1:1000	#2535S	Cell Signaling
**total-AMPK**	1:1000	#ab80039	Abcam
**Sirtuin 1**	1:1000	#8469	Cell Signaling
**p21 Waf1/Cip1 (p21)**	1:1000	#2947	Cell Signaling
**α-SMA**	1:500	#53-9760-80	Invitrogen
**CD31**	1:100	#ab28364<br>#14-0311-85	Abcam<br>Invitrogen
**CAV1**	1:1000	#3267	Cell Signaling
**CD34**	1:100	#553731	BD Biosciences
**peNOS (Ser1177)**	1:1000	#ab215717	Abcam
**eNOS**	1:1000	#32027S	Cell Signaling
**MPO**	1:200	#ab208670	Abcam
**F4/80**	1:100	#78538	Cell Signaling
**VEGF-A**	1:100	#ab46154	Abcam
**Endoglin (CD105)**	1:100	#ab221675	Abcam
**Biotinylated anti-mouse goat**	R.T.U.	#BP-9200	Vector Lab
**Goat anti-mouse Alexa 488**	1:500	#A11029	Thermo Fisher
**Goat anti-mouse Alexa 647**	1:500	#A21235	Thermo Fisher
**Goat anti-rabbit Alexa 647**	1:500	#A21244	Thermo Fisher

Table S2. qRT-PCR Primers and Their Sequences

Genes	Forward (5’→3’)	Reverse (3’→5’)
B55α	GAGACAAAAGACCAGAAGGCTATA	ATTCTCCGTGGACTGGCTTCCA
CAV1	CACACCAAGGAGATTGACCTGG	CCTTCCAGATGCCGTCGAAACT
Endoglin	CAGCCAAAGTGTGGCAATCAGG	GCTACTCAGGACAAGATGGTTCG
Epo	GACAAAGCCATCAGTGGTCTACG	GCAGAAAGTATCCACTGTGAGTG
Hgf	GTCCTGAAGGCTCAGACTTGGT	CCAGCCGTAAATACTGCAAGTGG
IL1β	GCCGTCTTTCATTACACAGG	CTCCACCTCAATGGACAGAA
IL6	GTATGAACAACGATGATGCACTTG	ATGGTACTCCAGAAGACCAGAGGA
IL10	GGTTGCCAAGCCTTATCGGA	ACCTGCTCCACTGCCTTGCT
IL12	GGAAGCACGGCAGCAGAATA	AACTTGAGGGAGAAGTAGGAATGG
PPIA	CATACAGGTCCTGGCATCTTGTC	AGACCACATGCTTGCCATCCAG
Sirt1	GGAGCAGATTAGTAAGCGGCTTG	GTTACTGCCACAGGAACTAGAGG
Vegf-A	CTGCTGTAACGATGAAGCCCTG	GCTGTAGGAAGCTCATCTCTCC

Supplementary Figures

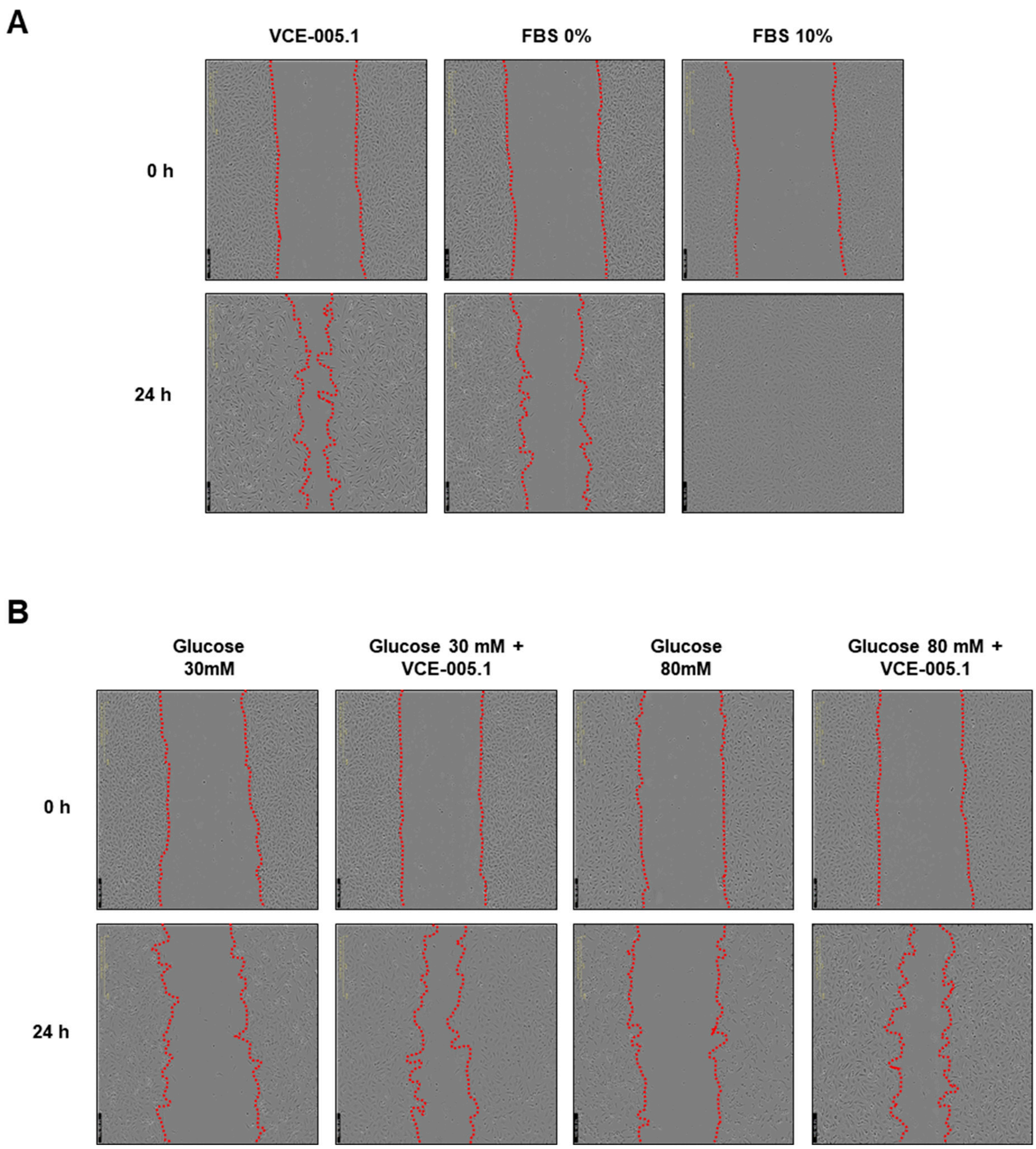

Figure S1. Bright-field images of endothelial wound closure following VCE-005.1 treatment under basal and hyperglycemic conditions. Representative bright-field images from the *in vitro* scratch wound healing assays shown in Figure 1B. **(A)** EA.hy926 cells were cultured in incomplete medium (0% FBS) and treated with VCE-005.1 (5 µM). Complete medium (10% FBS) was included as a positive control for wound closure. **(B)** EA.hy926 cells were exposed to 30 or 80 mM D-glucose for 24 h and subsequently treated with VCE-005.1 (5 µM) in incomplete medium. Images were acquired immediately after scratch induction (0 h) and after 24 h of treatment. Red dotted lines indicate the wound edges. Scale bars = 400 µm.

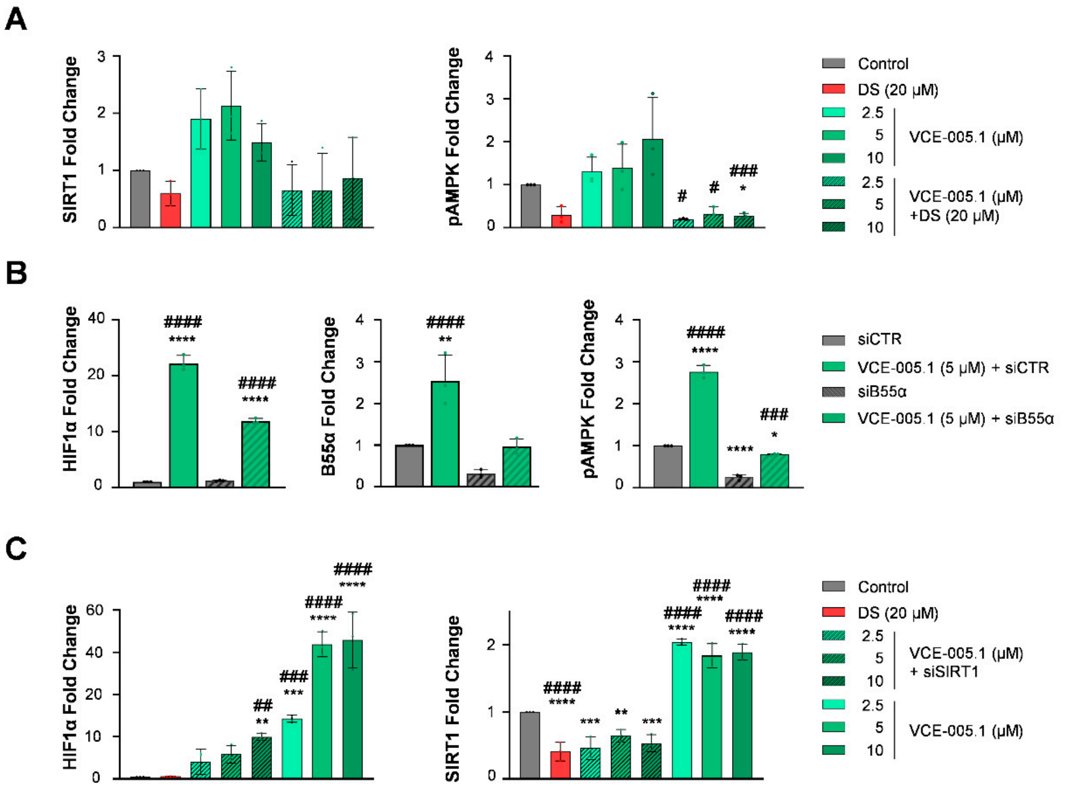

Figure S2. Signaling pathways involved in VCE-005.1-induced HIF-1α activation. (**A**) Quantification of SIRT1 and pAMPK protein expression from the Western blot experiments shown in Figure 2A. (**B**) Quantification of HIF-1α, B55α and pAMPK protein expression from the Western blot experiments shown in Figure 2B. (**C**) Quantification of HIF-1α and SIRT1 protein expression from the Western blot experiments shown in Figure 2C. Protein expression levels were normalized to β-actin or total AMPK, as appropriate. Data are presented as mean ± SD (n = 3 independent experiments). Normality was assessed using the Shapiro–Wilk test. Statistical significance was determined using one-way ANOVA followed by Dunnett's multiple comparisons test. Statistical significance is indicated in the panels as *p < 0.05, **p < 0.01, ***p < 0.001, and ****p < 0.0001 vs. Control cells; and #p < 0.05, ##p < 0.01, ###p < 0.001, and ####p < 0.0001 vs. DS-treated cells in (**A**), siB55α-transfected cells in (**B**), or siSIRT1-transfected cells in (**C**), as indicated in the panels.

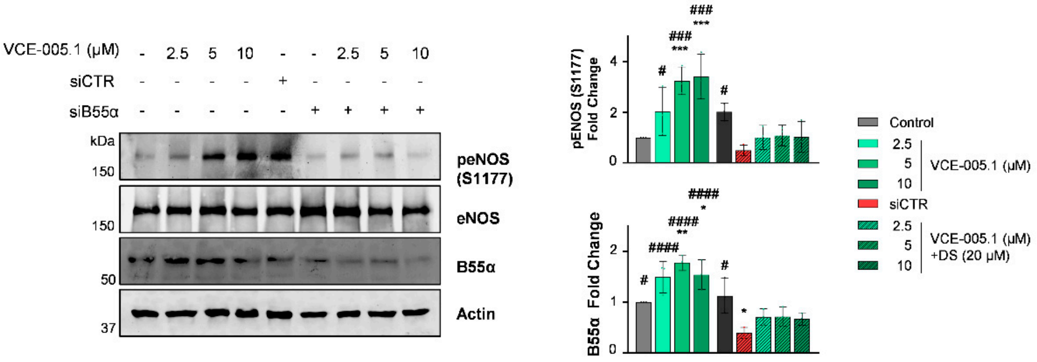

Figure S3. Effect of B55α silencing on VCE-005.1-induced eNOS phosphorylation. EA.hy926 cells were transfected with siB55α or scrambled control (siCTR) siRNA and subsequently treated with VCE-005.1 for 5 h. p-eNOS (Ser1177) levels were normalized to total eNOS, whereas B55α expression was normalized to β-actin. Data are presented as mean ± SD (n = 3 independent experiments). Normality was assessed using the Shapiro–Wilk test. Statistical significance was determined using one-way ANOVA followed by Dunnett's multiple comparisons test. Statistical significance is indicated in the panels as *$p < 0.05$, **$p < 0.01$, ***$p < 0.001$, and ****$p < 0.0001$ vs. control cells; and #$p < 0.05$, ###$p < 0.001$, and ####$p < 0.0001$ vs. siB55α-transfected cells, as indicated in the panels.

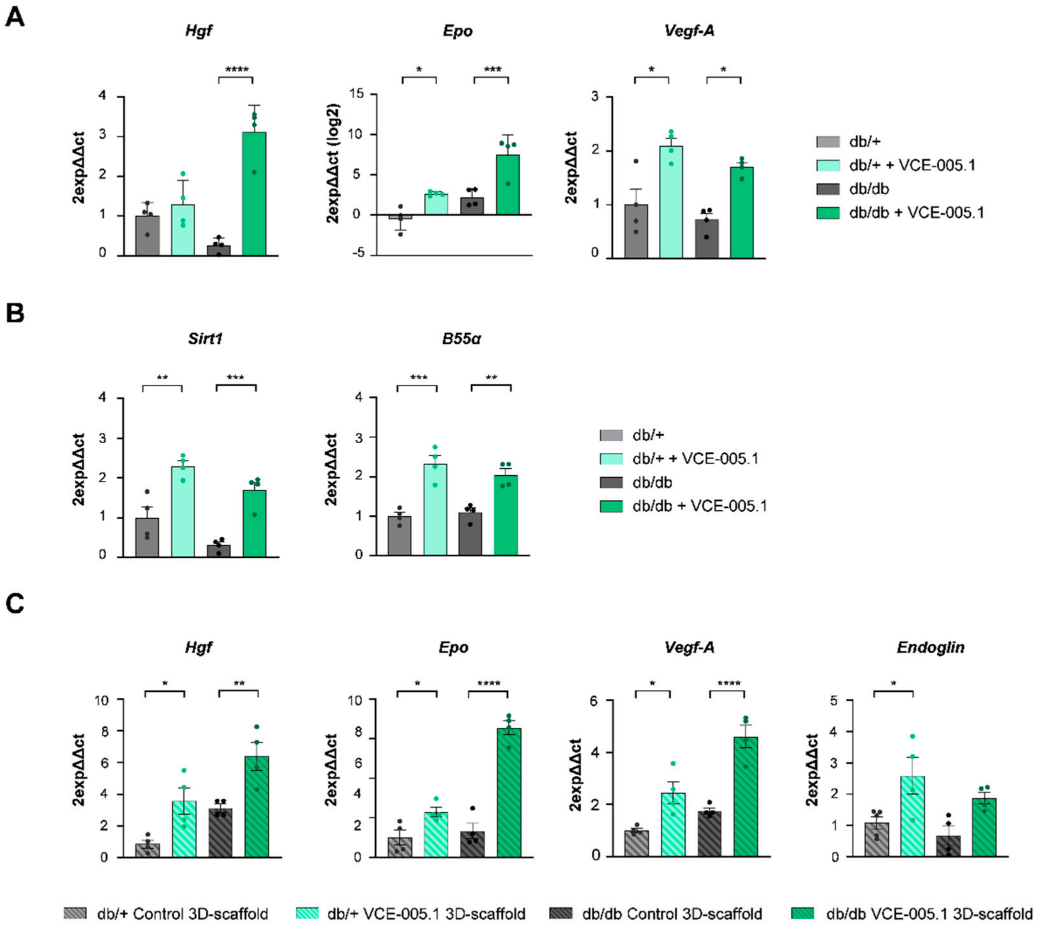

Figure S4. Gene expression of angiogenic and tissue repair markers in wound skin. (**A**) Relative mRNA expression of *Hgf, Epo,* and *Vegf-A* in wound skin collected 10 days after wounding following topical treatment. (**B**) Relative mRNA expression of *Sirt1* and *B55α* in wound skin collected 10 days after wounding following topical treatment. (**C**) Relative mRNA expression of *Hgf, Epo, Vegf-A,* and *Endoglin* in wound skin collected 14 days after treatment with control or VCE-005.1-loaded 3D-scaffolds. All results are presented as mean ± SEM (n = 4 animals per group). All datasets were assessed for normality using the Shapiro–Wilk test and analyzed using one-way ANOVA followed by Šídák's multiple comparisons test. Statistical significance is indicated in the corresponding panels as *p < 0.05, **p < 0.01, ***p < 0.001 and ****p < 0.0001.

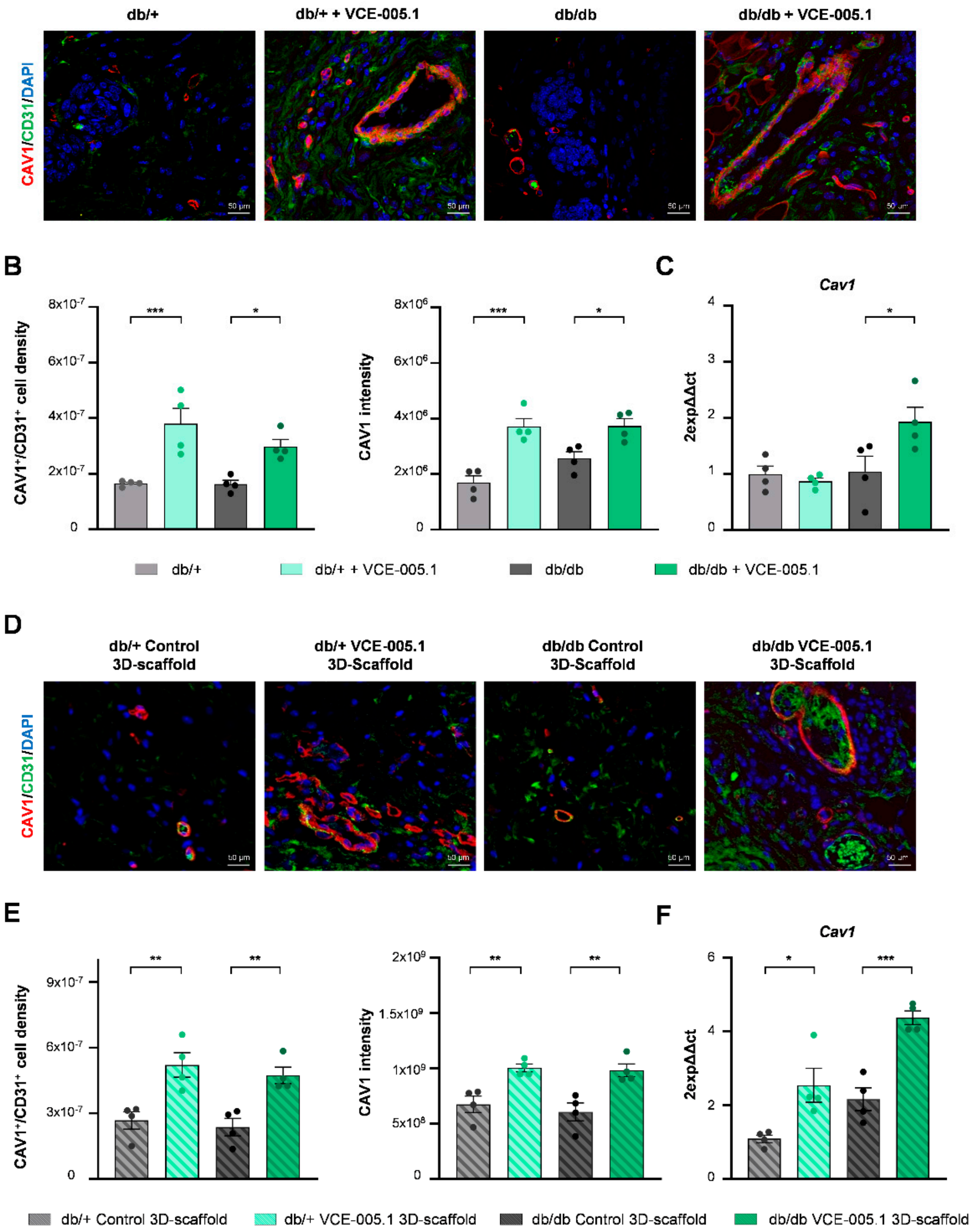

Figure S5. VCE-005.1 induces CAV1 *in vivo*. (**A**) Representative double immunofluorescence confocal staining of CAV1 (red)/CD31 (green)/DAPI (blue) in wound tissue. Scale bars = 50 µm; magnification = ×40. (**B**) Quantification of CAV1⁺/CD31⁺ cell density (cells/µm²) and CAV1 integrated fluorescence density. (**C**) Relative *Cav1* mRNA expression in wound skin collected 10 days after wounding following topical treatment. (**D**) Representative confocal double immunofluorescence staining of CAV1 (red)/CD31 (green)/DAPI (blue) in wound tissue from db/+ and db/db mice treated with control or VCE-005.1-loaded 3D-scaffolds. Scale bars = 50 µm; magnification = ×40. (**E**) Quantification of CAV1⁺/CD31⁺ cell density (cells/µm²) and CAV1 integrated fluorescence density. (**F**) Relative *Cav1* mRNA expression in wound skin collected 14 days after treatment with control or VCE-005.1-loaded 3D-scaffolds. Data are presented as mean ± SEM (for all experiments, n = 4 per group). Each data point represents one animal. All datasets were assessed for normality using the Shapiro–Wilk test and analyzed using one-way ANOVA

followed by Šídák's multiple comparisons test. Statistical significance is indicated in the corresponding panels as *p < 0.05, **p < 0.01 and ***p < 0.001.

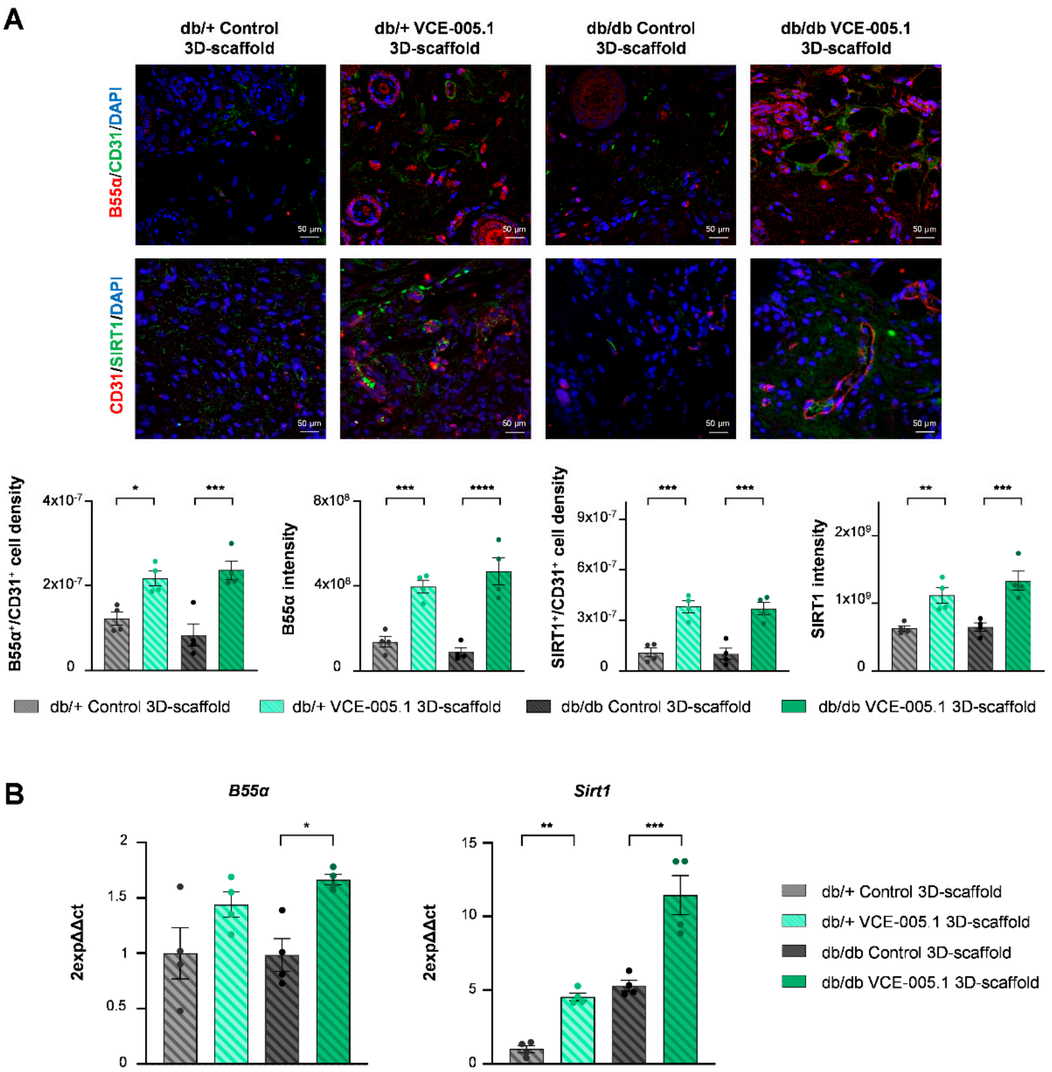

Figure S6. VCE-005.1 3D-scaffolds enhance B55α and SIRT1 expression during wound healing. (**A**) Representative confocal immunofluorescence images of wound sections stained for B55α (red), CD31 (green), and DAPI (blue), or SIRT1 (green), CD31 (red), and DAPI (blue), in db/+ and db/db mice treated with control or VCE-005.1-loaded 3D-scaffolds. $B55\alpha^{+}/CD31^{+}$ and $SIRT1^{+}/CD31^{+}$ cell densities (cells/μm^2), calculated as the number of double-positive cells divided by the corresponding tissue area analyzed, and B55α and SIRT1 integrated fluorescence densities were quantified. Scale bars = 50 μm; magnification = ×40. (**B**) Relative expression of *B55α* and *Sirt1* mRNA in wound skin 14 days after treatment with control or VCE-005.1-loaded 3D-scaffolds. Data are presented as mean ± SEM (n = 4 animals per group for all experiments). Each data point represents one animal. All datasets were assessed for normality using the Shapiro–Wilk test and analyzed using one-way ANOVA followed by Šídák's multiple comparisons test. Statistical significance is indicated in the corresponding panels as *p < 0.05, **p < 0.01, ***p < 0.001, and ****p < 0.0001.

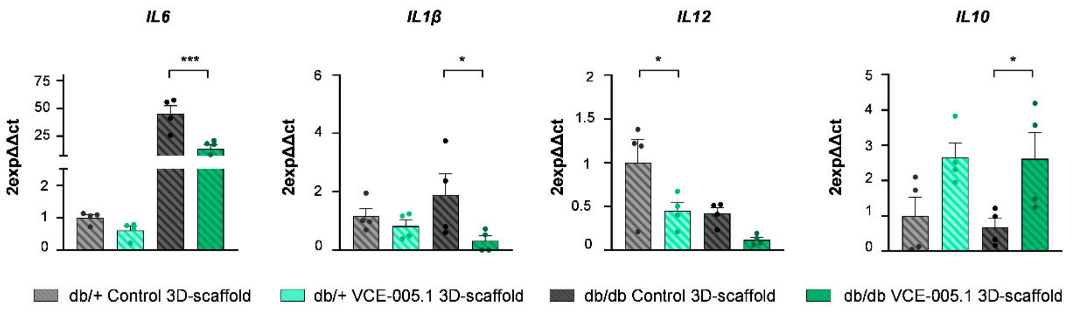

Figure S7. Effect of VCE-005.1-loaded 3D-scaffolds on inflammatory gene expression in wound tissue. Relative gene expression of *IL6*, *IL1β*, *IL12*, and *IL10* in wound tissue 14 days after treatment with control or VCE-005.1-loaded 3D-scaffolds. Data are presented as mean ± SEM (n = 4 animals per group). Each dot represents one individual animal. Normality was assessed using the Shapiro–Wilk test. Data were analyzed using one-way ANOVA followed by Šídák's multiple comparisons test, with significance indicated as *p < 0.05 and ***p < 0.001.
